# HarmonizR enables data harmonization across independent proteomic datasets with appropriate handling of missing values

**DOI:** 10.1038/s41467-022-31007-x

**Published:** 2022-06-20

**Authors:** Hannah Voß, Simon Schlumbohm, Philip Barwikowski, Marcus Wurlitzer, Matthias Dottermusch, Philipp Neumann, Hartmut Schlüter, Julia E. Neumann, Christoph Krisp

**Affiliations:** 1 Section Mass Spectrometry and Proteomics, Institute of Clinical Chemistry and Laboratory Medicine, University Medical Center Hamburg-Eppendorf (UKE), Hamburg, Germany; 2 High Performance Computing, Helmut Schmidt University, Hamburg, Germany; 3 Research Group Molecular Pathology in Neurooncology, Center for Molecular Neurobiology (ZMNH), University Medical Center Hamburg-Eppendorf, Hamburg, Germany; 4 Business division for Information Technology, University Medical Center Hamburg-Eppendorf, Hamburg, Germany; 5 Institute of Neuropathology, University Medical Center Hamburg-Eppendorf, Hamburg, Germany

**Keywords:** Proteomic analysis, Data integration

## Abstract

Dataset integration is common practice to overcome limitations in statistically underpowered omics datasets. Proteome datasets display high technical variability and frequent missing values. Sophisticated strategies for batch effect reduction are lacking or rely on error-prone data imputation. Here we introduce HarmonizR, a data harmonization tool with appropriate missing value handling. The method exploits the structure of available data and matrix dissection for minimal data loss, without data imputation. This strategy implements two common batch effect reduction methods—ComBat and limma *(removeBatchEffect()*). The HarmonizR strategy, evaluated on four exemplarily analyzed datasets with up to 23 batches, demonstrated successful data harmonization for different tissue preservation techniques, LC-MS/MS instrumentation setups, and quantification approaches. Compared to data imputation methods, HarmonizR was more efficient and performed superior regarding the detection of significant proteins. HarmonizR is an efficient tool for missing data tolerant experimental variance reduction and is easily adjustable for individual dataset properties and user preferences.

## Abstract (web summary)

Dataset integration is common practice to overcome limitations in statistically underpowered omics datasets. Here the authors present “HarmonizR”, a tool for missing data tolerant experimental variance reduction in large, integrated but independently generated datasets without data imputation, adjustable for individual dataset modalities, correction algorithm, and user preferences.

## Introduction

Omics experiments result in high dimensional data of different modality, including DNA-methylation profiles, the transcriptome, the proteome, and/or the metabolome. Regardless of the omics type, statistical validity of individually measured cohorts is often limited due to relatively small sample numbers. Data integration across multiple, individually conducted studies can efficiently increase cohort sizes. However, the integration of independent experimental settings requires the reduction of technically induced variances—so-called batch effects.

Commonly studied omics types, such as transcriptome and DNA-methylation data, are acquired on a limited number of platforms and inherit a relatively high comparability and data completeness across experiments. In these settings, batch effect reduction is well established^1,2^. In contrast, emerging technologies such as proteomics and single-cell-RNA sequencing suffer from low data completeness and high experimental variances across different quantification platforms and experimental setups^3,4^. To our knowledge, no large-scale data integration across independently generated proteomic datasets has been performed yet, while the topic of batch effect reduction has been addressed frequently for single cell RNA sequencing data^5^. Most of these tools are highly specific to single-cell RNA- sequencing data and cannot easily be adapted to other omics types (Sanorama^6^, scGene^7^, and Seurat^8^). Among the algorithms which are compatible with proteomic data, most strategies implement unsupervised non-linear dimension reduction methods such as principal component analysis (PCA) or t-distributed stochastic neighbor embedding (t-SNE) (Harmony^9^, LIGER^5^, deepMNN^10^, MMD-resnet^10^). Those standard multivariate exploratory methods use matrix algebra to produce an ordination of observations and require a complete data matrix by default. In contrast, limma’s *removeBatchEffect()* function depends on a linear regression model^11^. Finally, the most prominent tool—ComBat—is based on an empirical Bayes framework, enabling parametric and non-parametric batch effect reduction depending on, whether the user expects Gaussian or non-normally distributed data^12^. ComBat’s, as well as limma’s *removeBatchEffect()* function’s usage context, is limited to values missing in individual samples, whilst requiring features to be present in each batch. Hence, features not represented in all batches are excluded and datasets are restricted to common observations, dismissing valuable quantitative information.

Therefore, a major limitation when applying established batch effect reduction strategies to proteomic data is the inability of all approaches described above, to deal with missing values. Missing values are strongly represented in proteomic datasets and will be even more pronounced when integrating multiple cohorts or batches.

To bypass the missing value problem, data imputation may be applied^13,14^. However, the imputation of unknown scores based on existing values—frequently used in the context of batch effect reduction for proteomic data—is highly error prone^13^. Only truly missing scores, absent due to random effects can be imputed mathematically correct^15^. Quantitative information for peptides or proteins missing in an entire batch inherits a causality regarding the experimental setting or sample cohort. As shown for example by Cuklina et al., imputing these values for proteomic data can skew batch effects, resulting in incorrectly adjusted values and lead to false biological conclusions from batch-corrected proteomic datasets^16^. Therefore, the group integrated quantile and median normalization into their “*probatch”* package, disregarding more advanced approaches.

To overcome the lack of applicability of complex batch effect reduction algorithms to incomplete proteomic datasets, we developed the HarmonizR framework. HarmonizR allows handling of missing values by matrix dissection without the need for imputation or data reduction. In this framework, we integrated the two most predominately used, not on PCA-based methods for batch effect reduction—ComBat and the *removeBatchEffect()* function integrated in the limma package. Thus, HarmonizR enables missing data tolerant reduction of experimental variances in accordance with individual dataset properties and user preferences as a post processing step after database-driven protein identification. In the following, we show that HarmonizR-based integration of independent proteomic datasets, acquired from different tissue types, by different quantification strategies and instrumental setups is possible.

## Results

### The HarmonizR principle

**Fig. 1:**
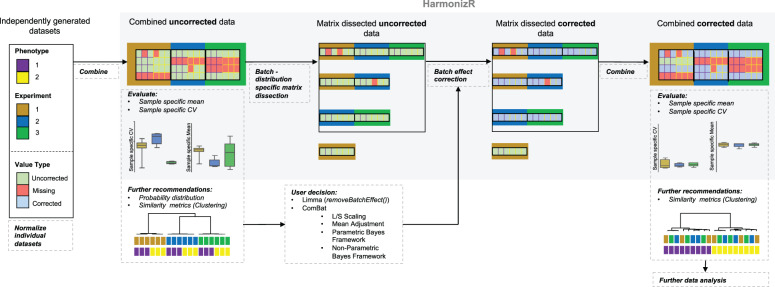
The general HarmonizR operation principle. Schematic representation of the HarmonizR operation principle for batch effect reduction across independent proteomic studies.

The HarmonizR framework is sketched in Fig. 1 (https://github.com/SimonSchlumbohm/HarmonizR). First, individual preprocessed datasets, which stem from different experiments, are combined in a matrix, including all samples and all proteins, that were detected in at least one batch. The core of HarmonizR is the missing value-dependent matrix dissection, that enables batch effect correction on sub-matrices. The algorithm is executed by calling the function *harmonizR()* (details on execution can be found in the HarmonizR SOP; see “Code availability” section). Initially, HarmonizR scans the input matrix for missing values. A batch is declared as missing if there are <2 values found for the respective protein. Sub-data frames are generated based on the batch count distribution of proteins. In a subsequent step, the selected batch effect correction method is executed for each sub-data frame. Proteins found in only one batch do not undergo harmonization. Based on the distribution of the data, the user can choose between limma’s *removeBatchEffect()* function or ComBat—the latter enabling parametric and non-parametric batch effect reduction—with or without scale adjustment. Finally, the corrected sub-matrices are merged to build up a ‘harmonized’, rejoined matrix (Fig. 1). Proteins found in only one batch are then added to this rejoined matrix.

When combining multiple datasets, missing values are introduced with each new batch. Therefore, the number of possible sub-matrices grows with the number of batches. For a dataset containing only two batches, three sub-matrices are built, of which the sub-matrix containing proteins appearing in both batches will be batch corrected. For three batches, there will be seven sub-matrices, of which ${t}_{3}=4$ sub-matrices contain proteins appearing in two or more batches. The maximum number of available combinations ${t}_{n}$ for batch-corrected sub-matrix construction given *n* batches, thus evolves naturally from

1 $${t}_{n}=\mathop{\sum }\limits_{k=2}^{n}\left(\begin{array}{c}n\\ k\end{array}\right)$$

with the usual binomial coefficient $({{n}\atop{k}})$. For example, when calculating the number of possible sub-matrices for 20 batches, the equation results in more than 10^6^ sub-matrices. However, this greatly exceeds the number of possible proteins/genes within a given dataset, in practice yielding a much lower upper limit. Moreover, in practice it is very unlikely for every batch combination to appear in a real-world scenario. Since the performance of HarmonizR is mostly dominated by the runtime and corresponding complexity of many independent ComBat/limma calls, these are executed in parallel within the HarmonizR framework to reduce processing time. The overall runtime and speed-up relative to the available cores can be viewed in Fig. 6 and Supplementary Fig. 1. Finally, the R-based HarmonizR strategy was also made available through Perseus—one of the most used software for statistical proteome analyses—by provisioning a Perseus plugin^17^ (Supplementary Information).

### HarmonizR versus imputation-based strategies

**Fig. 2:**
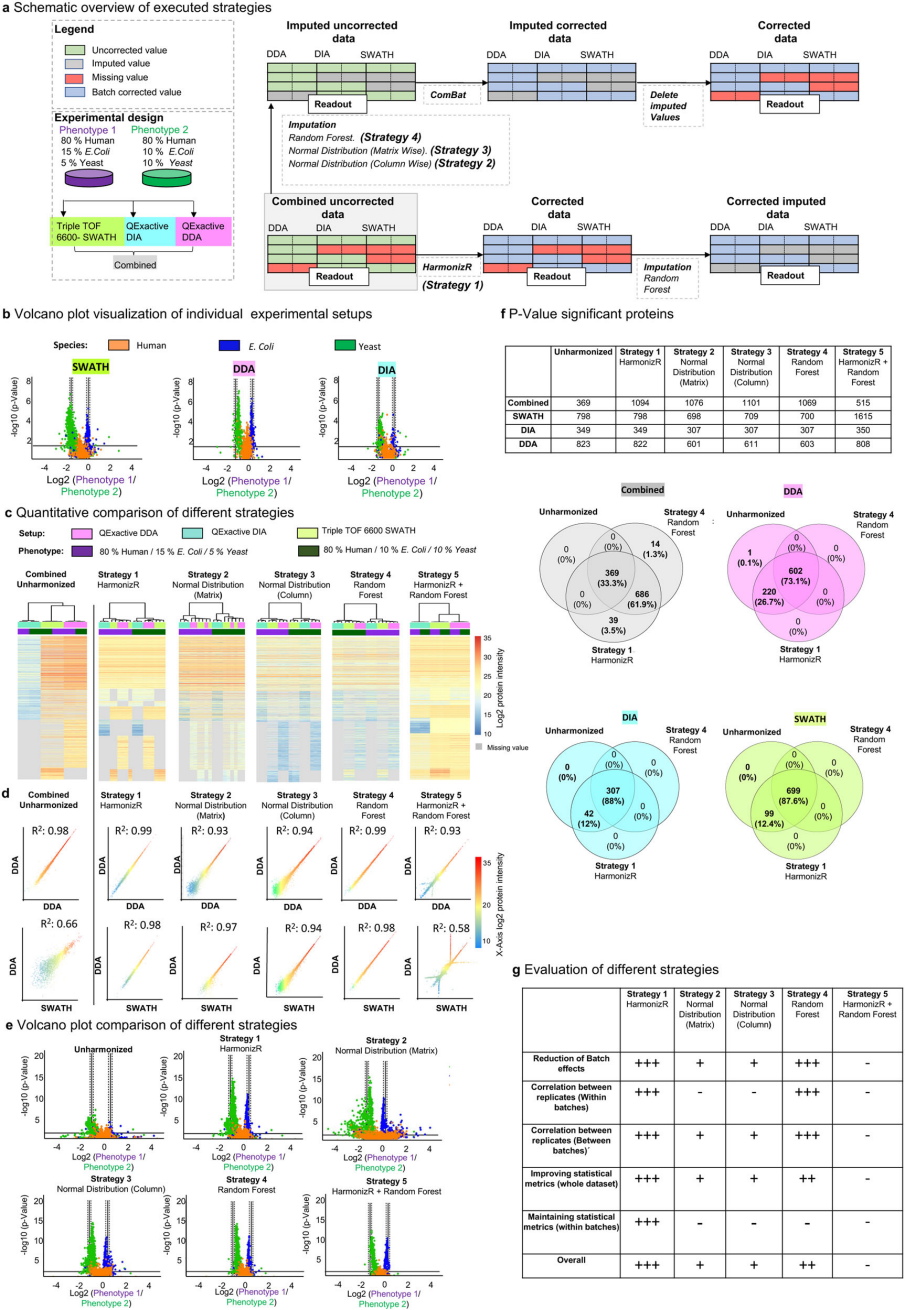
Comparison of HarmonizR to established missing value handling strategies for batch effect reduction. Comparison of imputation (Normal distribution -matrix wise, Normal distribution -column wise, Random Forest) to the HarmonizR framework, as missing value handling strategies for batch effect reduction, on K562 Chronic Myelogenous Leukemia cells spiked with 10% yeast 10% E. coli (phenotype 1) and 5% yeast and 15% E. coli (phenotype 2). Technical triplicates were measured in three different experimental setups (DDA data, acquired on a QExactive mass spectrometer, DIA data acquired on a QExactive mass spectrometer, and SWATH data acquired on a Triple TOF 6600 mass spectrometer). **a** Schematic overview of executed strategies. **b** Volcano plot visualization, plotting the log2 foldchange against the −log *p*-value for t-testing results between phenotype 1 and 2, for individual experiments (Two-sample Student’s T-test, *p*-value < 0.05). **c** Heatmap visualization of Pearson correlation-based hierarchical clustering with Ward.D linkage for all executed strategies. **d** Scatter plot visualization and corresponding correlalso made available through Persetion coefficient for phenotype 1 samples, measured with similar (DDA, upper panels) and different (DDA; SWATH, lower panels) LC-MS/MS setups for all executed strategies. **e** Volcano plot visualization, plotting the log2 foldchange against the −log *p*-value for t-testing results between phenotype 1 and 2 for combined data for all executed strategies proteins (Two-sample Student’s T-test, *p*-value < 0.05). **f** Number and overlap of *p*-value significant proteins (Two-sample Student’s T-test, *p*-value < 0.05), identified in t-testing between phenotype 1 and 2 for individual experiments and combined data for all executed strategies. **g** Evaluation of the suitability of executed imputation strategies and HarmonizR as missing value handling strategies for batch effect reduction. (−): Criteria not matched; (+) small improvement for respective criterion; (++) Improvement for respective criterion; (+++) Major improvement for respective criterion. Source data are provided as a Source Data file.

To test, whether technical batch effects caused by different LC-MS/MS instrumentation setups can be successfully reduced, we used a clearly defined setting first. Distinguishable phenotypes were generated by combining defined amounts of human, *E. coli*, and yeast cell lysates (Phenotype 1: 80% human, 10% *E. coli*, 10% yeast; phenotype 2: 80% human, 15% *E. coli*, 5% yeast, Fig. 2a). LC-MS/MS data on technical triplicates for each condition were acquired with three different LC-MS/MS setups (SWATH-Triple TOF 6600; DIA-QExactive, DDA-QExactive). To visualize the obtained protein fold changes between phenotype 1 and 2 for each of the three different quantification strategies, volcano plots showing the expected protein fold changes were generated (Fig. 2b). Combining all three datasets, only 34.62% of proteins (*n* = 1880) could be identified in all batches and an additional 1094 proteins (22.9%) were identified by at least two setups. Therefore, 2974 proteins required batch effect correction. In addition, 2569 Proteins were found in only one of the setups. As three different species result in a trimodal probability distribution of protein abundances, only the application of the non-parametric Bayes framework, implemented in the ComBat algorithm, was found to be applicable, as it does not presume a Gaussian probability distribution. For standard ComBat, at least one value in each batch is required for batch effect correction. Hence, 57.3% of the values must be omitted or imputed by artificial values.

To initially evaluate the applicability of HarmonizR (ComBat mode; non-parametric; L/S scaling) for missing value tolerant batch effect correction, the new framework (Strategy 1) was compared to

- ComBat after imputation of missing values from the normal distribution; matrix wise (Strategy 2);
- ComBat after imputation of missing values from the normal distribution; column wise (Strategy 3);
- ComBat after random forest (RF) imputation of missing values (Strategy 4);
- RF-based imputation of missing values after HarmonizR—based ComBat batch effect reduction (Strategy 5, Fig. 2a).

Hierarchical Clustering (HC) revealed a clustering according to the LC-MS/MS setup for combined, uncorrected data. The application of batch effect correction strategies 1–4 resulted in a clear distinguishability of phenotypes 1 and 2 (Fig. 2c), which was also observable after the usage of unmodified ComBat, based on 1880 proteins (Supplementary Fig. 2). RF imputation after HarmonizR (Strategy 5) reimplemented technical variances (Fig. 2c). To further investigate the impact of different strategies on the protein abundance distribution in individual samples, the correlation coefficient was calculated between similar samples measures with the same experimental setup (Phenotype 1; QExactive DDA vs QExactive DDA, Fig. 2d, upper panels, Supplementary Figs. 3–8) and different experimental methods (Phenotype 1; QExactive DDA vs Triple TOF 6600 SWATH, Fig. 2d). Before batch effect correction 99% linear correlation could be observed for the same setup (Fig. 2d and Supplementary Figs. 3–8). Between DDA and SWATH, the correlation coefficient dropped to 0.66 (Fig. 2d and Supplementary Figs. 3–8). For both Strategy 1 and 4, correlation coefficients between the same or different setups were higher (Fig. 2d and Supplementary Figs. 3–8). Moreover, the linearity reduced for low abundant proteins after batch effect correction for Strategies 2 and 3. For RF imputation after HarmonizR (Strategy 5), a skewed linear behavior could be observed (Fig. 2d and Supplementary Figs. 3–8). In all cases, batch effect correction led to a higher number of statistically significant classified yeast and *E. coli* proteins, combined with an evident decrease of *p*-values compared to individual experimental setups (Fig. 2b, e) and combined unharmonized data (Fig. 2e). Independent of the used strategy, the number of significant proteins, identifiable from DDA, SWATH, or DIA data alone changed after data harmonization, except for HarmonizR (Fig. 2f). In addition, more proteins could be correctly assigned to expected *p*-values and foldchanges between phenotype 1 and 2 (TN, TP) from integrated and harmonized data, while distribution patterns and quality of the original sub-data frames were retained. (Supplementary Fig. 10)

Log2 fold change differences between phenotype 1 and 2, on the other hand, were not significantly impacted by data combination and correction using Strategies 1, 3, 4 and 5 compared to DIA, DDA and SWATH observations alone. Only matrix wise imputation based on the normal distribution artificially increased abundance differences (Strategy 2, Fig. 2b, e). Of all strategies, only HarmonizR matched the expected difference of mean protein abundance (Supplementary Figs. 9, 10).

Overall, only HarmonizR (Strategy 1) and RF imputation prior to ComBat (Strategy 4) reduced batch effects, increased the correlation coefficient between samples that were acquired with different setups (while retaining the linearity between technical replicates) and sufficiently improved statistical metrics for combined data. However, HarmonizR was much easier to apply (no imputation steps and removal of artificial data necessary) and additionally maintained statistical metrics given in the original datasets individually for DDA, SWATH, and DIA (Fig. 2g).

### Harmonization across time points and tissue preservations

**Fig. 3:**
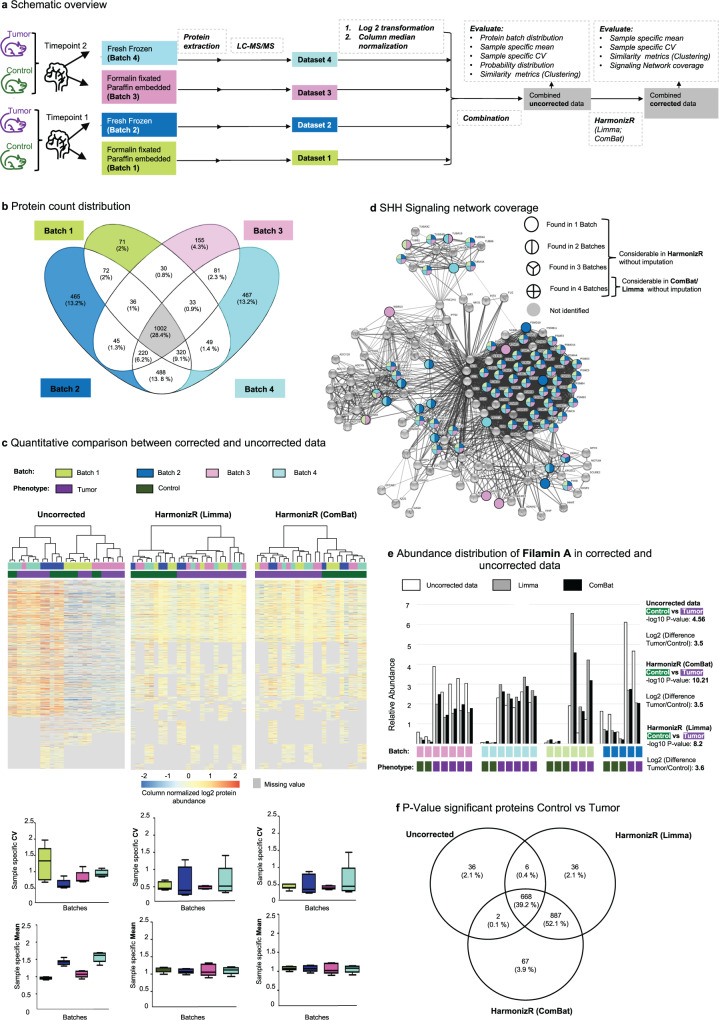
Limma and ComBat-based HarmonizR application for batch effect reduction across different experimental time points and tissue preservations. **a** Scheme of the experimental design. **b** Batch count distribution of all 3530 proteins quantified at least 2 times in a batch. **c** Heatmap visualization of Pearson correlation-based hierarchical clustering with Ward.D linkage for each tissue type and timepoint for unharmonized combined data, after ComBat- and limma- based HarmonizR execution. Sample specific CV and mean are shown on lower panels. (Batch 1 (green): *n* = 6 biologically independent animals (Tumor: *n* = 3; Control: *n* = 3); Batch 2 (blue): *n* = 5 biologically independent animals (Tumor: *n* = 2; Control: *n* = 3); Batch 3 (pink): *n* = 7 biologically independent animals (Tumor: *n* = 5; Control: *n* = 2); Batch 4 (turquoise): *n* = 7 biologically independent animals (Tumor: *n* = 5; Control: *n* = 2)). In boxplots, 50% of the data points are inside the box (Q1 (Quartile 1) being the lower bound of the box (25%), Q3 being the upper bound of the box (75%)). Whiskers show all values beyond the box without outliers. Outliners were defined as Q3 + 1.5 * IQR (Interquartile range) (upper outlier) and Q1-1.5 * IQR (lower outlier). IQR being Q1–Q3. **d** Batch specific coverage of proteins, associated with the “REACTOME-Signaling by Hedgehog” gene set. **e** Abundance distribution of the Sonic Hedgehog medulloblastoma Marker Filamin A in unnormalized data and after ComBat and limma- based HarmonizR execution. **f** Overlap of *p*-value significant proteins (*p*-value < 0.05), identified in t-testing between cerebellar tumors of hGFAP-cre::SmoM2^Fl/+^ mice and control cerebella in unnormalized data and after ComBat- and limma- based HarmonizR execution (Two-sample Student’s T-test, *p*-value < 0.05). Source data are provided as a Source Data file.

A predominate source of variation between proteomic experiments conducted in similar mass spectrometric setups are the tissue preservation method and the time of analysis. In order to test the reduction of these technical effects using limma’s *removeBatchEffect()* function or parametric ComBat in the HarmonizR framework, differently processed samples of an established Sonic hedgehog (Shh) medulloblastoma mouse model (*hGFAP-cre::SmoM2*^*Fl/+*^)^18^ were analyzed. In detail, cerebellar tumors of *hGFAP-cre::SmoM2*^*Fl/+*^ mice and control cerebella of littermate *SmoM2*^*Fl+*^ mice were bisected. One half was snap frozen at −80 °C (fresh frozen (FF) condition). The other half was Formalin fixated, and paraffin-embedded (FFPE condition). Experiments were repeated to vary the time of analysis (timepoint 1 and 2, Fig. 3a), resulting in four technical batches. Performing separate database searching for each batch, to mimic independently generated studies, 3530 proteins were identified, whilst only 1002 (28.4%) were identifiable in all batches. 1374 proteins (38.9%) were additionally identified in at two or three batches, while 1154 candidates (32.7%) were found in one batch (Fig. 3b) exclusively. Thus, 71.6% of the data was not subjectable to unmodified limma/Combat without imputation. HC revealed a batch-based clustering for combined, uncorrected data (Fig. 3c). As shown for unmodified limma and ComBat alone, (Supplementary Fig. 11), batch effect reduction, using HarmonizR (ComBat/limma) corrected the data and resulted in a clear distinguishability of the phenotypes; in this case tumor and control, while considering a significantly higher number of proteins. At the same time no remaining separation based on tissue type or preparation timepoint was observable (Fig. 3c). Despite equal distribution of phenotypes across batches, the sample-specific mean and coefficient of variance (CV) differed prior to normalization (Fig. 3c, lower left panels). As activated Shh signaling is driving Shh medulloblastoma growth, we looked at coverage of the Shh signaling network (Fig. 3d). Only 20 out of 71 identified proteins (28%) could be considered by standard strategies. In contrast, HarmonizR was able to consider 100% of the Shh signaling pathway-associated proteins that were found by LC-MS/MS in at least one batch (Fig. 3d).

To further evaluate the impact of batch effect reduction on individual proteins, the abundance distribution of the Shh medulloblastoma biomarker Filamin (FLNA)^19^ was assessed prior to and after batch effect adjustment. While a 11-fold higher FLNA abundance could be detected in tumor samples prior to and after HarmonizR usage, the −log (*p*-value) significantly increased (Fig. 3e).

Of note batch effect reduction was also required when datasets were processed together from raw LC-MS/MS files, allowing. Here, missing values are rescued by aligning and matching peaks across different LC-MS/MS runs while non-biological variances are reduced by chromatographic alignment. Using the Minora Algorithm, 4786 proteins were quantified in total. While a higher data completeness (75.8% of all proteins were found in all batches) was obtained 24.8% of all identified proteins required HarmonizR-based matrix dissection for batch effect reduction. (Supplementary Fig. 12).

Comparing HarmonizR (limma) to HarmonizR (Combat), only slight differences could be observed. Both techniques significantly reduced technical variances. Between tumor and control mice, 887 additional proteins showed significant t-test differences for both algorithms compared to uncorrected data. However, only a small number of candidates was considered significant after using HarmonizR with a specific algorithm (ComBat: 67 Proteins, limma: 36 proteins) (Fig. 3f).

Next, we tested the applicability of a HarmonizR-based strategy for data harmonization across different quantification platforms. Therefore, a dataset on “spike-in stable isotope labeling by amino acids in cell culture” (spike-in SILAC), “Tandem Mass Tag” (TMT), and “Data depended acquisition” (DDA) mode “Label free quantification” (LFQ) quantified proteins, published by Stepath et al. in 2019^20^ was used.

The dataset contained data on HiFi cells with or without cetuximab treatment after 0, 3, and 24 h. Data acquisition was performed in quadruplicates. In this work, the authors describe a distinguishability of the 24 h treated condition from all other conditions for spike-in SILAC and LFQ quantified proteins. This effect could not be observed based on TMT quantified proteins. Prior to comprehensive data harmonization of TMT, spike-in SILAC, and LFQ data, quantification technique-specific data normalization was performed for individual datasets. Spike-in SILAC and LFQ data were normalized by column-wise median subtraction. TMT-based quantification of four TMT 8-plexes required internal TMT batch adjustment prior to quantification strategy-based adjustment. According to Stepath et al., internal reference scaling (iRS) was carried out at the protein level, based on two reference samples, integrated in each batch, to reduce TMT batch effects^20^.

As iRS depends on the usage of internal references, TMT data cannot be integrated independently of the conducted experiment. Furthermore, the use of individual samples for batch effect adjustment is very prone to experimental inaccuracies.

**Fig. 4:**
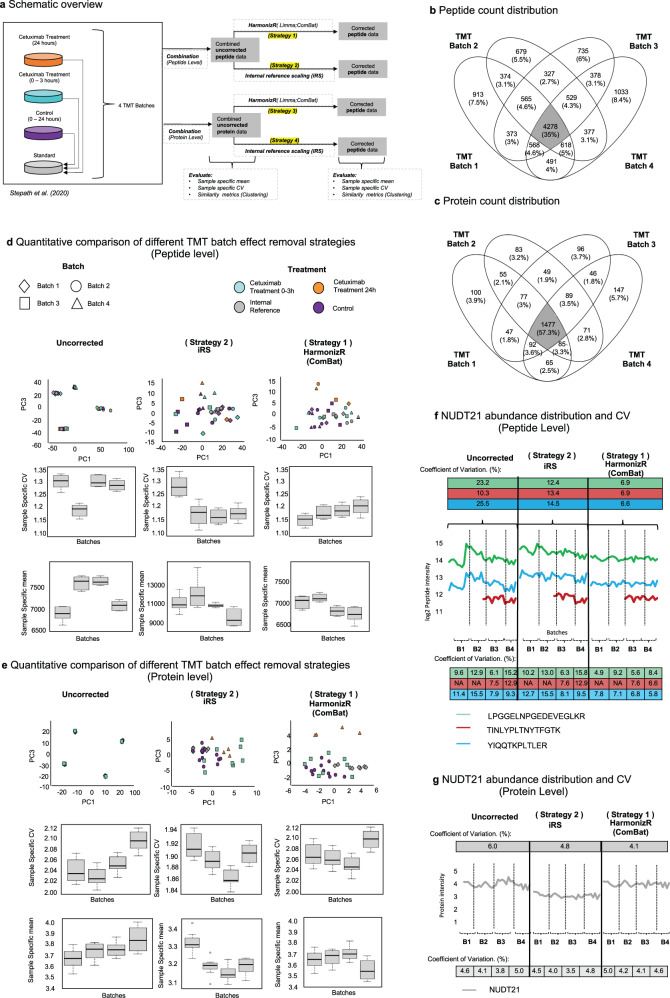
Comparison of HarmonizR and iRS for the batch effect reduction in Multiplex TMT experiments at the peptide and protein level. **a** Experimental design. **b** Batch count distribution of 12615 peptides quantified. **c** Batch count distribution of 2579 proteins quantified. **d** Scatter plot distribution of samples across PC1 and PC3 in NIPALS-PCA, based on 8877 peptides found in 50% of all samples and corresponding sample specific CV/means for each TMT batch for unnormalized data, after iRS normalization and after HarmonizR (ComBat). (All batches represented *n* = 8 biologically independent samples. (Control: *n* = 3; Cetuximab 24 h: *n* = 1; Cetuximab 0–3 h: *n* = 3; Internal reference: *n* = 2). In boxplots, 50% of the data points are inside the box (Q1 (Quartile 1) being the lower bound of the box (25%), Q3 being the upper bound of the box (75%)). Whiskers show all values beyond the box without outliers. Outliners were defined as Q3 + 1.5 * IQR (Interquartile range) (upper outlier) and Q1-1.5 * IQR (lower outlier). IQR being Q1–Q3. **e** Scatter plot distribution of samples across PC1 and PC3 in NIPALS-PCA, based on 2152 proteins found in 50% of all samples and corresponding; sample specific CV/means for each TMT batch for unnormalized data, after iRS normalization and after HarmonizR (ComBat). (All batches represented *n* = 8 biologically independent samples. (Control: *n* = 3; Cetuximab 24 h: *n* = 1; Cetuximab 0–3 h: *n* = 3; Internal reference: *n* = 2). In boxplots, 50% of the data points are inside the box (Q1 (Quartile 1) being the lower bound of the box (25%), Q3 being the upper bound of the box (75%)). Whiskers show all values beyond the box without outliers. Outliners were defined as Q3 + 1.5 * IQR (Interquartile range) (upper outlier) and Q1-1.5 * IQR (lower outlier). IQR being Q1–Q3. **f** Overall and batch-specific variation of specific peptides for the housekeeping protein NUDT21 for unnormalized data, after iRS normalization, and after HarmonizR (ComBat). **g** Overall and batch-specific variation of NUDT21 for unnormalized data, after iRS normalization and after HarmonizR (ComBat) at protein level. Source data are provided as a Source Data file.

Therefore, the applicability of HarmonizR was evaluated for TMT data and compared to iRS at the peptide and protein level (Fig. 4a).

### Comparing HarmonizR to iRs for TMT Batch effect reduction

In total, 12615 peptides, corresponding to 2579 proteins, were identified. 35% of peptides and 57.3% of the proteins were found in all 4 TMT batches (Fig. 4b, c).

As a Gaussian probability distribution and divergent sample-specific mean and CV could be observed at peptide and protein level, L/S scaling within ComBat’s parametric Bayes Framework was used as an example to be compared to iRS (Fig. 4d, e).

Missing value tolerant, “Nonlinear Iterative vertical Least Squares (NIPALS)” PCA^21^ was carried out to compare dataset variances before normalization, after iRS and after ComBat HarmonizR considering multiple dimensions. Prior to batch effect reduction, samples clustered in dependence of their respective TMT batch. For iRS, batch effects were still visible in principle components (PCs) 1 to 3 (Fig. 4d, e; Supplementary Fig. 13). For all calculated PCs, no separation of 24 h cetuximab treated cells was observable. According to Stepath et al., no clear distinguishability of the divergent condition “24 h cetuximab treatment” could be obtained for TMT data (in contrast to spike-in SILAC and LFQ data, see also Supplementary Fig. 14)^20^. Using HarmonizR, batch effects were efficiently reduced. Additionally, 24 h cetuximab treated samples were clearly distinguishable in PC 3, for peptide and protein data. At both levels, the best possible approximation of sample-specific means and CVs was observable after HarmonizR usage (Fig. 4d, e).

To evaluate the effect of all applied strategies on an individual protein, the overall and batch-specific coefficient of variation was calculated for the protein Nuxid Hydrolase 21 (NUDT21). NUDT21 has been described previously as a housekeeping protein for proteomic analysis as it showed the lowest CV across 27 different tissue types (4.9%) represented in the human proteomic database (ProteomicDB)^22^.

At the peptide level, the overall CV was exemplary evaluated for 3 unique peptides of NUDT21. For all peptides, HarmonizR reduced the CV to 6–7%, while for iRS normalized peptides, CVs between 12 and 15% were observable (Fig. 4f). At the protein level, an overall CV of 6% was observed prior to batch effect reduction. After iRS, the CV across all batches decreased to 4.8% while HarmonizR-based batch effect reduction resulted in the lowest CV across all batches with 4.1% (Fig. 4g).

**Fig. 5:**
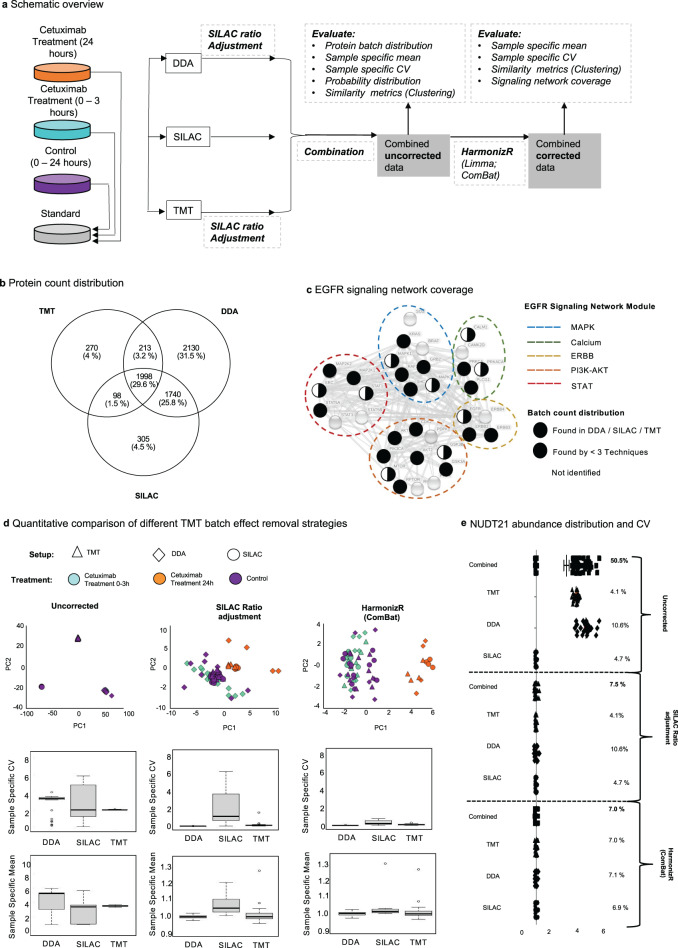
Data harmonization across different quantification approaches (DDA, TMT, and SILAC) for Cetuximab treated HiFi cells. **a** Scheme of the experimental design. **b** Batch count distribution of 6754 proteins quantified. **c** Batch-specific coverage of proteins, associated with the EGFR signaling network (Stepath et al., 2020). **d** Scatter plot distribution of samples across the top two principal components (PC) in NIPALS-PCA, based on 2368 proteins, found in 50% of all samples for unnormalized data after SILAC ratio adjustment and data after HarmonizR (ComBat). Lower panels show corresponding sample-specific CV and mean values. (DDA: *n* = 24 biologically independent samples (Control: *n* = 12; Cetuximab 24 h: *n* = 4; Cetuximab 0–3 h: *n* = 8); TMT: *n* = 24 biologically independent samples (Control: *n* = 12; Cetuximab 24 h: *n* = 4; Cetuximab 0–3 h: *n* = 8); SILAC: *n* = 23 biologically independent samples (Control: *n* = 12; Cetuximab 24 h: *n* = 4; Cetuximab 0–3 h: *n* = 7); TMT: 32 biologically independent samples (Control: *n* = 12; Cetuximab 24 h: *n* = 4; Cetuximab 0–3 h: *n* = 7; Internal reference: *n* = 8)). In boxplots, 50% of the data points are inside the box (Q1 (Quartile 1) being the lower bound of the box (25%), Q3 being the upper bound of the box (75%)). Whiskers show all values beyond the box without outliers. Outliners were defined as Q3 + 1.5 * IQR (Interquartile range) (upper outlier) and Q1-1.5 * IQR (lower outlier). IQR being Q1–Q3. **e** Overall and batch specific variation of the housekeeping protein NUDT21 for unnormalized data, after SILAC ratio adjustment and after HarmonizR (ComBat) usage. Source data are provided as a Source Data file.

Hence, ComBat HarmonizR enabled the distinguishability of the 24 h Cetuximab condition and revealed a low overall CV for housekeeping proteins. Therefore, HarmonizR adjusted protein data was used as a basis for data integration across different quantification approaches (Fig. 5).

### Harmonization across different quantification techniques

Combining TMT, spike-in SILAC and LFQ data resulted in 6754 quantified protein groups, with 1998 (29.6%) being detected by all techniques (Fig. 5a, b). Focusing on the Cetuximab impacted EGFR signaling network defined by Stepath et al.^20^, 26 out of 34 factors were quantified in total, while only 9 proteins were found in DDA, spike-in SILAC, and TMT data at the same time (Fig. 5c).

For spike-in SILAC data, relative protein quantities are displayed as ratios between each individual sample and a labeled reference. To align TMT and DDA data to spike-in SILAC data, individual ratios between a respective protein and its mean abundance across all samples were calculated (“SILAC ratio adjustment”, Fig. 5a, d). Based on sample-specific mean and CV values as well as a Gaussian probability distribution of protein abundances, L/S scaling within ComBat’s parametric Bayes framework was exemplarily applied within HarmonizR (Fig. 5a, d). NIPALS PCA (Fig. 5d) revealed, that prior to SILAC ratio-based data alignment, differences between TMT, DDA and spike-in SILAC were obvious (67% of all differences explained in PC1), suppressing phenotypical differences. After DDA and TMT data alignment to spike-in SILAC-like ratios, PCA revealed a general distinguishability of 24 h Cetuximab treated samples, while quantification technique dependent effects were still observable. After additional ComBat HarmonizR-based data harmonization, the technical batch effect was efficiently reduced, while 24 h Cetuximab treated samples formed a distinct cluster (Fig. 5c). Unsupervised hierarchical clustering confirmed these results (Supplementary Fig. 14). Prior to data harmonization, different sample-specific mean and CV values were observed for each quantification platform (Fig. 5d). Spike-in SILAC samples showed the lowest sample-specific CV. However, the highest variance among sample specific CVs could be observed. After SILAC ratio adjustment, sample specific CV and mean values were significantly reduced for TMT and DDA data. HarmonizR-based batch effect reduction resulted in comparable values across all quantification platforms, while a slightly higher variance across sample-specific CV values within the spike-in SILAC data set remained (Fig. 5d).

Furthermore, the intra-quantification platform and overall CV for the housekeeping protein NUDT21 were calculated. Prior to inter-quantification platform normalization, the overall CV was 50.5% (Fig. 5e). TMT samples and spike-in SILAC samples showed a CV of 4.1 and 4.7%, respectively. The highest intra-study CV was observable for DDA data with 10.6%. SILAC ratio adjustment of TMT and DDA data reduced the overall CV to 7.6%, while quantification technique specific CVs were retained. ComBat HarmonizR-based data harmonization reduced the overall and quantification technique-specific CV for NUDT21 to 6.9–7.0% (Fig. 5e).

### Run time optimization for large datasets

**Fig. 6:**
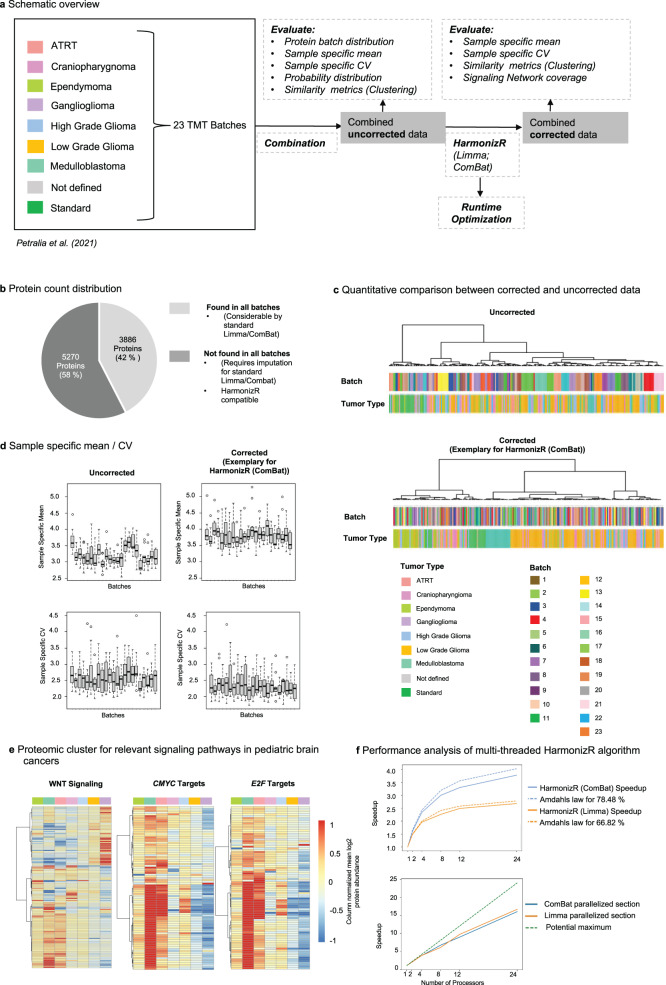
Application and performance optimization of HarmonizR for large datasets based on different Brain Tumor entities, measured in 23 TMT batches^23^. **a** Schematic representation of the experimental design. **b** Batch count distribution of 9156 proteins quantified. **c** Pearson correlation-based hierarchical clustering with Ward.D linkage prior to and after HarmonizR (ComBat) usage. **d** Sample specific CV and mean for uncorrected data and after HarmonizR (ComBat) execution. (All batches consisted of *n* = 11 biologically independent samples, details on the assignment of tumor types to batches can be obtained from Supplementary Table 5). In boxplots, 50% of the data points are inside the box (Q1 (Quartile 1) being the lower bound of the box (25%), Q3 being the upper bound of the box (75%)). Whiskers show all values beyond the box without outliers. Outliners were defined as Q3 + 1.5 * IQR (Interquartile range) (upper outlier) and Q1-1.5 * IQR (lower outlier). IQR being Q1–Q3. **e** Heatmap visualization of tumor type specific abundance distribution of proteins, associated with the tumor relevant gene sets “Hallmark-MYC Targets; Hallmark-E2FTargets and REACTOME -Signaling by WNT” after HarmonizR (ComBat) execution. **f** Performance analysis of multi-threaded HarmonizR algorithm. 1.: Visualization of the speedup of the HarmonizR implementation for ComBat (blue) and limma (orange) alongside Amdahl’s law (dashed lines) with respect to the number of processors. Tests have been made for 1, 2, 4, 8, 12, and 24 processors. Code corresponding to 78.48% of the sequential run time has been parallelized for ComBat and 66.82% for limma. Amdahl’s law has therefore been calculated using these percentages. 2.: Speedup visualization for the parallelized part of the HarmonizR implementation only with respect to the number of processors. Speedup while using the ComBat algorithm is shown in blue. Speedup while using the limma algorithm is shown in orange. Tests have been made for 1, 2, 4, 8, 12, and 24 processors. The potential maximum speedup is shown as a linear, proportional behavior. Computed on an Intel Xeon Gold 6226, 2.70 GHz, 2 × 12 compute cores, 96 GB RAM. Source data are provided as a Source Data file.

Finally, we analyzed the applicability of the HarmonizR framework for datasets, with a higher number of batches. Therefore, a dataset published by Petralia et al. in 2021, comparing proteomic profiles across eight different pediatric brain tumor entities, measured in 23 TMT eleven-plexes was used (Fig. 6a)^23^. In total, 9156 proteins were quantified, and 3886 proteins were found in all batches (Fig. 6b). As a Gaussian probability distribution and divergent sample-specific mean and CV could be observed, L/S scaling within ComBat’s parametric Bayes framework was used as an example (Fig. 6d). Pearson correlation-based HC showed the high similarity between respective batches prior to HarmonizR usage (Fig. 6c). HC after HarmonizR-based batch effect reduction revealed a clear differentiability of medulloblastoma and ependymoma, while more mixed clusters are obtained for example for low grade glioma (LGG), high grade glioma (HGG), and ganglioglioma (Fig. 6c). To further compare the findings proposed by Petralia et al. to HarmonizR batch-corrected data, we evaluated the mean abundance of proteins associated with predominant cancer pathways (MYC, E2F, and WNT). Especially MYC and E2F associated proteins showed a significantly higher abundance in ATRT and medulloblastoma. These results go in line with the findings, proposed by Petralia et al.^23^ (Fig. 6e).

Matrix dissection of 23 batches may potentially result in 4194281 combinations of submatrices for batch correction (see equation above), however, 3654 submatrices occurred in the experiment. The measured speedup is shown for both the entire HarmonizR execution (with visualization of Amdahl’s law^24^) and the parallelized part only. Both HarmonizR variants (limma and ComBat) are considered (Fig. 6f). The study reveals that parallelizing the execution of ComBat and limma is sufficient to improve the performance of the program by a factor 2 (limma)/factor 3 (ComBat) at workstation level. This behavior is in line with strong scaling theory as indicated by the trend predicted with Amdahl’s law.

## Discussion

With the HarmonizR framework, we present a tool capable of handling omics datasets with missing values of random and not random type, that is flexibly applicable in a variety of settings at the post protein identification level, independent of the availability of spectral data.

The tool enables data harmonization across batches without the need for data reduction or imputation, as it performs data dissection into batch distribution-specific sub-data frames for batch effect adjustment. Integrating HarmonizR with ComBat and limma^11,25^, the strategy can equally be applied to Gaussian or non-normally distributed data. However, in principle the HarmonizR framework can be combined with any algorithm with batch effect reduction capabilities, having a potential to be suitable for any data modalities and diverse scientific questions.

For technical highly variable proteome data, we specifically showed for the first time that efficient batch effect reduction without data imputation or massive data loss is feasible. Moreover, we established a HarmonizR plugin for the most frequently used software for proteome analyses—Perseus^17^—which facilitates the usage of this tool for a broad spectrum of users. All packages as well as the plugin are publicly available (see data availability). Based on four analyzed datasets we show that efficient reduction of batch effects is possible at the peptide as well as the protein level. This applies to technical variations across different tissue preservation techniques, different LC-MS/MS instrumentation setups, and quantification approaches, including batch effect reduction across Tandem Mass Tag (TMT)-plexes.

Here, HarmonizR was found to be beneficial for the identifier-based combination of protein abundances after individual database searching for individual studies as well as for combined database searching of LC-MS/MS data from different cohorts, aiming to rescue missing values and reduce technical variances by chromatographical alignment. (Fig. 3; Supplementary Fig. 12).

The matrix dissection approach comes with an increasing amount of function calls as the number of batches within the data rises. As the number of created sub-data frames grows rather quickly, we parallelized ComBat/limma *removeBatchEffect()* function calls, supporting their simultaneous execution on both shared-memory (i.e., personal computers/notebooks) and distributed-memory (i.e., cluster computer) systems. Nevertheless, the upper limit of created sub-data frames described by the binomial coefficient is not expected to occur, as several housekeeping proteins are likely to occur in all batches, and submatrices are limited by absolute number of proteins detected. This leads to significantly shorter execution times than theoretically expected. The parallel execution calls improve the performance of the HarmonizR algorithm and allow computation of larger datasets if provided with the appropriate computational power.

To date, there are only few proteome data-specific tools that allow for batch effect reduction. Most tools addressing this problem—such as ProNorM—require negative controls or internal standards to reduce unwanted technical variations^26^. Thus, they are not compatible with the integration of independently generated proteomic datasets stored at online repositories that do not contain similar samples.

Recently, a first step to adjust for batch effects in these proteomic datasets was made, implementing basic quantile and median normalization in tools like “proBatch”^16^. As a limitation, more advanced strategies, routinely used for other types of omics studies would be more beneficial but are highly limited due to the high number of missing values in proteomic datasets^16^.

Most RNA sequencing/transcriptome-based strategies for batch effect reduction, compatible with the structure of proteomic data, rely on unsupervised non-linear dimension reduction methods such as PCA or t-distributed stochastic neighbor embedding (t-SNE), that require a complete data matrix by default (Harmony^9^, LIGER^5^, deepMNN^10^, MMD-resnet^10^). Hence, these algorithms are not suitable for datasets with missing values.

Modified versions of t-SNE and PCA, such as InDaPCa are applicable but are currently not implemented in the respective batch effect reduction algorithm structure^27^.

The most prominent approaches, limma’s *removeBatchEffect()* function that implements a linear regression model^11^, and ComBat, which is based on an empirical Bayes framework^25^, can deal with missing at random data but are not applicable when a protein is missing in an entire batch. Of note, in the four datasets examined in this study, we found, that between 42.7 and 71.6% of all proteins were missing in at least one of the included batches. Thus, reducing these datasets to proteins compatible with existing batch effect reduction strategies is associated with a significant loss of relevant biological information. This was exemplarily shown for an Shh-signaling network^28^ in a mouse medulloblastoma dataset and EGFR network for Cetuximab stimulated DiFi cells^20^.

Incomplete proteomic data is especially evident when the data-dependent acquisition (DDA) principle is used, where the identification of peptides is determined by their experimental setup dependent peptide environment^29^. Hence, we found that this problem became more evident the more different the experimental setups of individual batches were. When combining four TMT 8-plexes (Stepath et al. 2020), 42.7% of proteins were found missing in at least one of the four batches. In comparison to that, combining different experimental setups, a significant smaller proportion of proteins was found in all batches (Mixed organism proteome measured with DDA-QExactive, DIA-QExactive, and SWATH-Triple TOF 6600 data: 34.62%; murine medulloblastoma samples (FFPE and FF tissue) at two different experimental timepoints: 28.4%; Cetuximab stimulated DiFi cells and controls, quantified by DDA-LFQ, spike-in SILAC or TMT: 29.6%).

To tackle these problems, we linked the HarmonizR framework to limma’s *removeBatchEffect()* function as well as the ComBat algorithm to enable advanced batch effect reduction resulting in minimal data loss.

For all analyzed types of technical variances, limma’s *removeBatchEffect()* or ComBat based HarmonizR, successfully reduced batch effects (different mass spectrometers; different quantification approaches; different tissue types). This was evident since samples that previously clustered according to the technical setup formed phenotype- based clusters after the application of HarmonizR in all cases. These results were comparable to the application of unmodified ComBat (Supplementary Figure 2) but considered a significantly higher number of proteins. Furthermore, expected biological characteristics were correctly represented after batch effect reduction. This is for example indicated by the protein abundance distribution of the well-known cancer signaling pathways MYC and E2F^30^, which is in line with the findings described by Petralia et al.^23^. In addition to the increasing correlation between similar samples measured with different techniques, HarmonizR was also able to sustain similarity metrics within individual batches. Hence, the number of *p*-value significant proteins in the mixed organism proteome dataset increased from 369 to 1094 (Fig. 2). At the same time, the number of *p*-value significant proteins between both conditions remained constant, when testing each batch separately.

As an alternative to data reduction, imputation may be applied to bypass missing values prior to the usage of ComBat or limma^23^. Depending on the used strategy, the imputation of values is highly error-prone^15^. This is especially evident when missing at random (MAR = proteins, missing in individual samples) and missing not at random (MNAR = proteins, missing in entire batches) type missing values are present at the same time^31^.

In this case, we compared HarmonizR implemented ComBat to the usage of ComBat after executing different missing value imputation techniques frequently used in literature^23^, such as “imputation from the normal distribution”^17^ or “random forest imputation”^32^.

When applying imputation strategies, we found random forest-based imputation superior to normal distribution-based imputation, which is well in line with the literature^32^ (Fig. 2). For both, random forest imputation and HarmonizR, batch effects were successfully reduced, correlation coefficients between biological replicates within the same batch were retained and the correlation between phenotypical replicates of different batches increased after batch effect reduction. However, only the HarmonizR strategy retained significant differential abundant proteins within individual batches whereas this piece of information is partially lost when using the random forest-based strategy. These findings agree with previous studies. The imputation, of MNAR type missing values resulted in incorrectly adjusted values that altered the number and identity *p*-value of significant proteins and thus lead to false biological assumptions^16^. This is particularly problematic when the number of missing values of the “not at random” type for a protein was greater than 8.5%^13^, which is given for all proteins missing in only one batch in this study.

HarmonizR was found to be superior to the imputation-based handling of missing values (prior to batch effect reduction). In case, a complete data matrix is mandatory for subsequent statistical data analysis steps—despite the danger that artificially introduced values distort biological statements—imputation followed by batch effect adjustment is preferable to imputation after batch effect correction. We saw that the latter reimplemented batch effects and skewed protein abundances as well as statistical metrics among and within different batches.

Removing technical variances between proteomic experiments, the experimental setup, and the type of variance has a large impact on how data should be preprocessed. This was especially important when integrating different label-free and isotope label-based quantification techniques.

We demonstrated for example, that spike-in SILAC data could only be integrated with TMT and LFQ quantification strategies after quantitative values for each protein from TMT and LFQ data were normalized to their mean abundance across all samples, mimicking the ratio between a sample set representative labeled control and a respective sample. Following this procedure, a successful batch effect reduction was demonstrated for the combined dataset of Stepath et al.^20^ with distinguishability of the 24 h Cetuximab treated samples. The latter could previously be shown for individual datasets^20^.

Furthermore, the evident decrease of the overall CV for the housekeeping protein Nuxid Hydrolase 21 (NUDT21) after data harmonization to an expected value < 10%^22^ underlined a successful batch effect reduction.

Thus, ComBat HarmonizR successfully enabled data harmonization across different mass spectrometric setups and quantification platforms and therefore allows for a combined analysis of independently generated proteomic datasets for biomarker discovery research.

The HarmonizR principle was also shown as an effective batch effect reduction strategy across different TMT plexes and outperforms the internal reference scaling (iRS) approach commonly used for TMT batch effects. HarmonizR-based data harmonization outperformed iRS as the reduction of technical biases at the peptide and protein level was more efficient. In contrast to iRS it accounted for varying mean sample specific CVs in phenotypically equal distributed TMT batches (Variance scaling) and did not require identical internal reference standards to reduce batch effects. This facilitates experimental design and makes it possible to integrate independently generated TMT batches. While ComBat has been used previously to compensate for TMT batch effects in single studies^23,33,34^, the ComBat HarmonizR strategy now allows integration of different TMT-plexes without imputation and data reduction. This is especially important for large patient cohorts, as with each integrated TMT batch data completeness, concordance and reproducibility is significantly reduced^35^.

In summary, the matrix dissection approach of the HarmonizR algorithm and its fundamental idea to abstain from calculating or imputing any missing values itself, can be adapted for the adjustment of any type of omics data while assuring minimal data loss. This will make integration of proteomic LC-MS/MS data sets in online repositories or previously generated in-house datasets possible and can overcome sample number limitations in proteome-based discovery studies.

## Methods

### Publicly available datasets

To test the applicability of HarmonizR for the batch effect reduction between different quantification approaches, a dataset published in 2021 by Stepath et al. was used^20^. In brief, 0, 3, and 24 h Cetuximab treated DiFi cells and untreated controls were measured using spike-in stable isotope labeling by amino acids in cell culture (spike-in SILAC); Tandem Mass Tag (TMT) and DDA -based Label Free Quantification (LFQ), respectively, on a Quadrupole—Orbitrap Hybrid mass spectrometer (QExactive, Thermo Fisher Scientific). The dataset can be accessed via the PRIDE archive (PXD014565).

The usability of HarmonizR for bigger datasets, including multi-threaded runtime optimization, was tested on a dataset measured by Petralia et al. in 2021^23^. Concisely seven different brain tumor entities were distributed over twenty-three TMT 10-plex batches and measured using a Orbitrap-Iontrap-Quadrupol Tribrid mass spectrometer (Orbitrap Fusion, Thermo Fisher Scientific)^23^. The dataset is available through the Clinical Proteomic Tumor Analysis Consortium Data Portal (https://cptac-data-portal.georgetown.edu/cptacPublic/) and the Proteomics Data Commons (https://pdc.cancer.gov/pdc/).

### Experimental setup for in house generated datasets

For the comparison of HarmonizR to established missing value handling strategies, an artificial mixed organism proteome with expected foldchange differences was generated by combining defined amounts of human K562 Chronic Myelogenous Leukemia cell digests (Promega), *Escherichia Coli* (donated by Prof. Holger Rohde, University Medical Center Hamburg Eppendorf, Institute of Microbiology) and *Saccharomyces Cerevisiae* (Promega) digests. Three technical replicates of 80% human cell digests; 10% E. Coli; 10% yeast and 80% human; 15% E. Coli; 5% yeast were measured using three different mass spectrometric setups (DDA- nano-UPLC (nano-Acquity, Waters) coupled to an Orbitrap Hybrid mass spectrometer (QExactive, Thermo Fisher Scientific); Data independent acquisition (DIA)- nano-UPLC (nano-Acquity, Waters) coupled to an Orbitrap Hybrid mass spectrometer (QExactive, Thermo Fisher Scientific); SWATH- UPLC (Dionex UltiMate 3000, Thermo Fisher Scientific) coupled to an Quadrupole time of flight MS (TripleTOF 6600, SCIEX)). For further details on protein extraction, tryptic digestion, and LC-MS/MS setups, please refer to the PRIDE archive (PXD027467).

To evaluate HarmonizR-based batch effect reduction between different tissue types (Formalin fixated paraffin-embedded (FFPE); fresh frozen (FF)) and experimental timepoints, a medulloblastoma mouse model was used. *hGFAP-cre::SmoM2*^*Fl/+*^ tumors and cerebella of *SmoM2*^*Fl+*^ littermate controls at postnatal day 13 were analyzed. Both male and female mice were used. *hGFAP-cre* mice^36^ and *SmoM2*^*Fl/Fl*^ mice^37^ were purchased from The Jackson Laboratories (Bar Harbor, ME, USA). Transgenic mice were bred with a C57BL/6J background. All experiments using animals were approved by the local animal care committee (Behörde für Justiz und Verbraucherschutz in Hamburg, TVA N99/2019) and handling was conducted in accordance with local governmental and institutional animal care regulations. Cerebellar tumors of *hGFAP-cre::SmoM2*^*Fl/+*^ and cerebella of *SmoM2*^*Fll+*^ littermate controls were bisected. One half was snap frozen and stored at −80 °C degrees until further processing (fresh frozen (FF) condition). The other half was fixed in 4% paraformaldehyde/PBS overnight at room temperature. Tissue for paraffin-embedded sections was dehydrated, embedded, and sectioned at 4 μm according to standard protocols (FFPE condition). Histomorphology of tumor or cerebellar tissue was verified by H&E-staining.

For proteomic measurements, DDA-based LFQ was used on a nano-UPLC (Dionex UltiMate 3000, Thermo Fisher Scientific), coupled to an Orbitrap-Iontrap-Quadrupol Tribrid mass spectrometer (Orbitrap Fusion). For further details on protein extraction, tryptic digestion, and LC-MS/MS setups, please refer to the PRIDE archive (PXD027467).

### Raw data processing

DDA raw spectra, from mixed proteome and medulloblastoma mice samples were processed with the Andromeda algorithm, implemented in the MaxQuant software (Max Plank Institute for Biochemistry, Version 1.6.2.10). All batches were searched separately to mimic independently generated datasets. For the mixed organism proteome, spectra were searched against the reviewed human database (Downloaded from Uniprot December 2017, 26559 entries), yeast (Saccharomyces cerevisiae strain ATCC 204508, downloaded from Uniprot December 2017, 6721 entries), and *Escherichia Coli* (Strain K12, downloaded from Uniprot December 2017, 31400 entries). Mouse tissue samples were searched against a reviewed murine database (downloaded from Uniprot December 2020, 17015 entries). Trypsin was selected as enzyme used to generate peptides, allowing a maximum of two missed cleavages. A minimal peptide length of 6 amino acids and maximal peptide mass of 6000 Da was defined. Oxidation of methionine, phosphorylation of serine, threonine and tyrosine, acetylation of protein N-termini, and the conversion of glutamine to pyro-glutamic acid was set as variable modification. The carbamidomethylation of cysteines was selected as fixed modification.

For mixed proteome samples, the error tolerance for the first precursor search was 20 ppm. 10 ppm was applied for mouse samples. For the following main search, 4.5 ppm was used in all experiments. Fragment spectra were matched with 20 ppm error tolerance. A false discovery rate (FDR) value threshold <0.01, using a reverted decoy peptide database approach was set for peptide identification. Label-free quantification was performed with an LFQ minimum ratio count of 1. Matching between commonly searched runs was applied to increase data completeness.

HarmonizR is designed as a post-processing tool for the integration and harmonization of independently generated datasets, independent of the availability of LC-MS/MS raw data. To highlight the advantage of combined raw data processing of different batches, prior to HarmonizR usage, additional combined database searching was performed for medulloblastoma mice samples. Here, Sequest combined with the Minora algorithm^38^, implemented in Proteome Discoverer 2.4 (Thermo Fisher Scientific) was used to rescue missing values and reduce non biological variances across LC-MS/MS runs, by chromatographic alignment.

For DIA data, acquired DDA LC-MS/MS data of mixed proteomes as well as DDA runs of human yeast and *E. coli* digests only were used to generate a reference peptide spectra library for data extraction and searched against the reviewed human, yeast, and *E. coli* protein database, using the Sequest algorithm integrated in the Proteome Discoverer software version 2.0 (Thermo Fisher Scientific). Mass tolerances for precursors were set to 10 ppm and 0.02 Da for fragments. Carbamidomethylation was set as a fixed modification for cysteine residues and the oxidation of methionine, pyro-glutamate formation at glutamine residues at the peptide N-terminus as well as acetylation of the protein N-terminus, methionine loss at the protein N-terminus and the acetylation after methionine loss at the protein N-terminus were allowed as variable modifications. Only peptide with a high confidence (FDR <1% using a decoy data base approach) were accepted as identified. Proteome Discoverer search results were imported into Skyline software version 4.2, allowing only high confidence peptides with more than 4 fragment ions. A maximum of 5 fragment ions per peptide were used for information extraction from DIA files for peptides with a dot product of >0.85. Peptide peak areas were summed to generate protein areas which were then used for relative abundance comparison.

### Normalization and combination of individual datasets

Obtained relative intensities for protein groups were loaded into the Perseus software (Max Plank Institute for Biochemistry, Version 1.5.8.5) for each experiment and batch separately for preparative normalization prior to HarmonizR usage. Processed data from individual batches was combined based on the UniProt identifier. The resulting combined datasets were subjected to HarmonizR-based batch effect reduction.

For the mixed organism proteome dataset, prior to batch effect reduction between data SWATH, DIA and DDA data, relative protein abundance values were log2 transformed. Due to the trimodal probability distribution of *E. coli* and yeast spiked K562 Chronic Myelogenous Leukemia cell lines, no further normalization was performed.

For medulloblastoma mouse models, relative protein abundances were log2 transformed and median normalized across columns, prior to batch effect reduction between FFPE and FF data acquired at different experimental timepoints.

For Cetuximab treated DiFi cells (Stepath et al. 2021) TMT, spike-in SILAC, and DDA data was handled differently. Initially, batch effects were reduced between TMT eight plex batches. Therefore, TMT reporter intensities were log2 transformed and median normalized across columns. Normalized TMT reporter intensities were subjected to HarmonizR. For performance comparison to internal reference scaling (iRS), the adjustment of TMT batches was additionally performed by dividing reporter ion intensities by the arithmetic means of the two channels representing the reference mix, as described by Stepath & Zülich et al.^20^. Based on the results, only HarmonizR adjusted data was subjected to further processing steps. DDA and SILAC datasets were log2 transformed and median normalized across columns. To mimic spike-in SILAC ratios, normalized DDA and TMT datasets, mean subtraction was applied across rows prior to data combination.

For brain tumor samples (Petralia et al. 2021), TMT reporter intensities were log2 transformed and median normalized across columns prior to batch effect reduction between TMT 11-plexes.

### Usage of the HarmonizR algorithm

HarmonizR was built as a framework to enable the handling of missing values without the need for imputation, using the ComBat algorithm or the “*removeBatchEffect()*” function implemented in the limma package in the R software environment.

The algorithm is executed by calling the function “*harmonizR()”*. After reading the input, HarmonizR sorts every feature into its corresponding sub-data frame based on the number of existing numerical values within each batch. A feature is discarded for a certain batch if there are <2 values present, based on the chosen parameters. Each sub-data frame is then individually batch effect adjusted by the chosen adjustment algorithm. The “*removeBatchEffect()*” function from limma (https://rdrr.io/bioc/limma/man/removeBatchEffect.html)^11^ fits a linear model to the data, including both batches and regular treatments, then removes the component due to the batch effects. ComBat (https://rdrr.io/bioc/sva/man/ComBat.html)^12^ enables the usage of parametric or nonparametric empirical Bayes framework models for batch effect reduction. Batch effect reduced sub-data frames are rejoined and an output file is generated. HarmonizR optionally visualizes the sample-specific mean, feature specific mean or Coefficient of variation (CV) for each batch prior to and after data adjustment to enable direct visual evaluation of the executed batch effect reduction. For further information about the application of HarmonizR, please refer to https://github.com/SimonSchlumbohm/HarmonizR.

### Application of the HarmonizR framework for batch effect reduction on different datasets

All datasets, excluding the mixed organism proteome dataset, were processed in the HarmonizR framework using the parametric Bayes framework with L/S adjustment, integrated in the ComBat algorithm and the “*removeBatchEffect()*” function in limma, assuming a Gaussian probability distribution.

For mixed organism proteome samples, a trimodal probability distribution is given. Therefore, the non-parametric empirical Bayes framework with L/S adjustment, integrated in the ComBat algorithm, was applied for the reduction of batch effects across different mass spectrometric setups.

### Data imputation for mixed organism proteome samples

For the comparison of HarmonizR to established missing value handling strategies, three different imputation strategies were applied to the mixed organism proteome dataset prior to batch effect reduction. 1. Matrix and 2. Column wise imputation from the normal distribution: was carried out in Perseus with a width of 0.3 and a value downshift of 1.8. Random Forest imputation was performed using the “RandomForest”package implemented in the R software environment. Imputation was performed in an unsupervised mode, using 1000 trees in 10 iterations. Similar settings for Random Forest imputation were used to evaluate the applicability of imputation after HarmonizR usage.

In line with the HarmonizR-based strategy, the non-parametric Bayes framework, implemented in the Combat algorithm was used for batch effect reduction after missing value imputation.

### Statistical data analysis and visualization

All t-tests, integrated in this study were carried out using the Perseus software (Max Plank Institute for Biochemistry, Version 1.5.8.5). Proteins, identified with a *p*-value < 0.05 between respective testing conditions, were considered as statistically significant differentially abundant. Pearson correlation-based hierarchical clustering with Ward.D linkage was performed using the “pheatmap” package (Version 1.0.12) in the R software environment (Version 4.0.4) Missing value tolerant Non-Linear Iterative Square (NIPALS) PCA was performed using the NIPALS-PCA function integrated in the “mixomics” package^39^ (Version 6.20.0). Scatter plot distributions of samples across principal components were visualized in PRISM (GraphPad, Version 5). Venn diagrams were generated using Venny (BioinfoGP, Version 2.1.0). Abundance distributions of individual proteins were visualized using Microsoft Excel (Version 16.5.).The genesets “REACTOME – SHH Signaling”; “REACTOME -WNT Targets Hallmark -MYC Targets” and “Hallmark -E2F Targets” were obtained from the Molecular Signature Database (https://www.gsea-msigdb.org/gsea/msigdb/). To support the visualization of the EGFR and Shh signaling Network the STRING Protein–Protein interaction database was used (https://string-db.org). Boxplots were been created using the R built-in function *boxplot()*. The visualization of the speedup has been done in the Python programming language (Version 3.8.) using the “matplotlib” package (Version 3.5.2.)^40^.

### Reporting summary

Further information on research design is available in the Nature Research Reporting Summary linked to this article.

## Supplementary information

Supplementary Information

Reporting Summary

## Data Availability

All raw and processed proteomic data generated in this study has been deposited to ProteomeXchange Consortium via PRIDE, under the accession code PXD027467. Publicly available datasets, used in this study, can be accessed via PRIDE, under the accession code PXD014565 (Stepath et al.^20^ (https://pubs.acs.org/doi/full/10.1021/acs.jproteome.9b00701)) (https://www.ebi.ac.uk/pride/archive/projects/PXD014565) or through the Clinical Proteomic Tumor Analysis Consortium Data Portal (https://cptac-data-portal.georgetown.edu/cptacPublic/) under the accession code PDC000204 (Petralia et al.^23^ (https://pubmed.ncbi.nlm.nih.gov/33242424/)), respectively. Source data are provided with this paper.

## Source data

Source Data

## Code availability

The HarmonizR package and the Perseus plugin, including detailed descriptions can be accessed via GitHub: https://github.com/SimonSchlumbohm/HarmonizR (10.5281/zenodo.6553171. Additionally, the HarmonizR package will also be available on Bioconda.
