# Supplementary material for: HarmonizR enables data harmonization across independent proteomic datasets with appropriate handling of missing values
Source: Nat Commun. 2022 Jun 20;13:3523. doi: 10.1038/s41467-022-31007-x (PMC9209422; doi:10.1038/s41467-022-31007-x)
Supplement: Supplementary file 1 — Supplementary Information [file 41467_2022_31007_MOESM1_ESM.pdf]

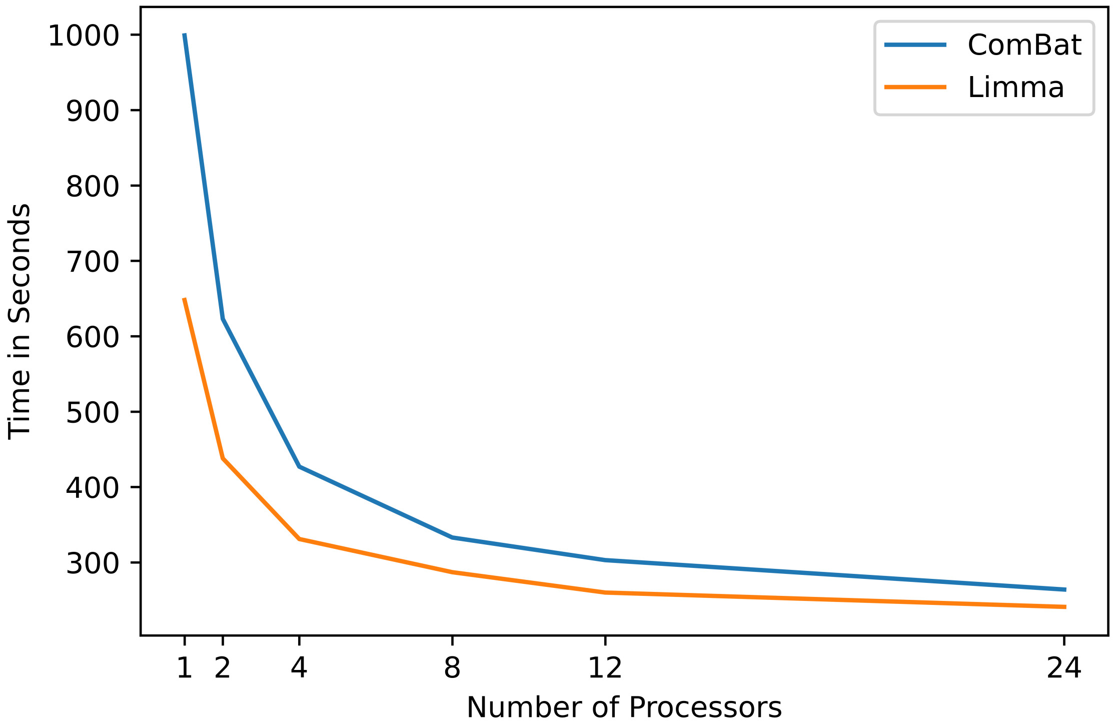

Supplementary Figure 1: Visualization of the runtime in seconds of the HarmonizR implementation by using ComBat (blue) and Limma (orange) with respect to the number of processors*. Tests have been made for 1, 2, 4, 8, 12 and 24 processors. The analysis has been performed on the dataset published by Petralia et al. (2021).*

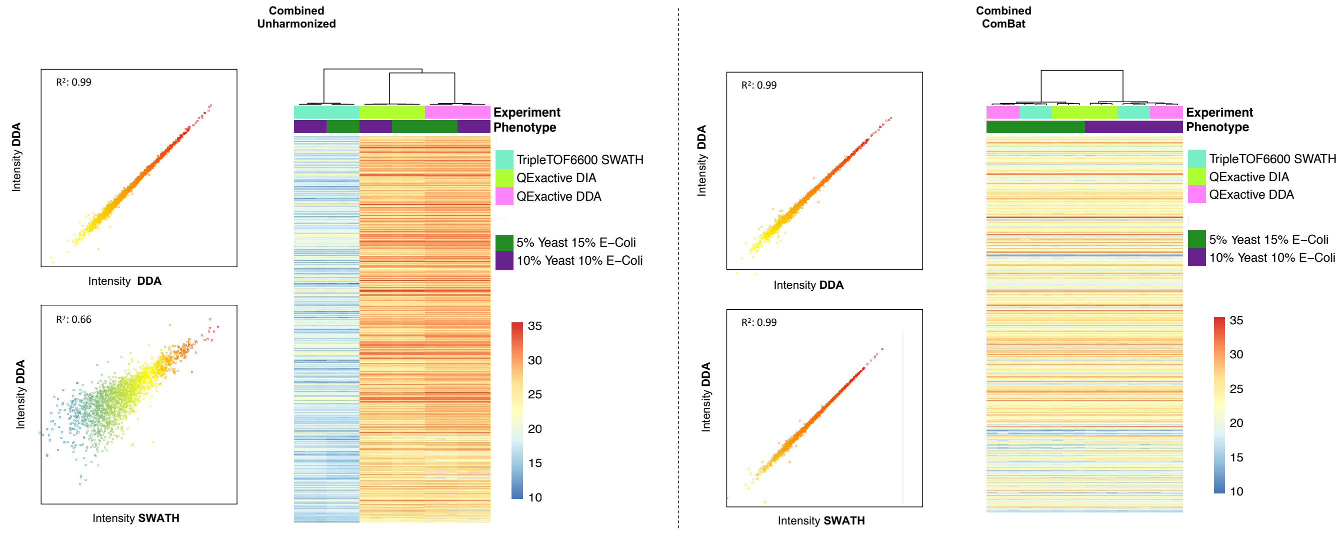

Supplementary Figure 2: Quantitative comparison of unharmonized and ComBat processed data on K562 Chronic Myelogenous Leukemia cells spiked with 10 % yeast 10 % E. coli (phenotype 1) and 5 % yeast and 15 % E. coli (phenotype 2), measured with different LC-MS/MS setups (Triple TOF6600 SWATH; QExactive DIA; QEcaxtive DDA). *1880 proteins that do not inherit missing not at random (MNAR) type missing values and therefore are compatible with the unmodified ComBat algorithm were considered. Unnormalized, log2 transformed data was used. The nonparametric empirical bayes framework, implemented in the ComBat algorithm was used for batch effect reduction including R/S scaling. Source data are provided as a Source Data file.*

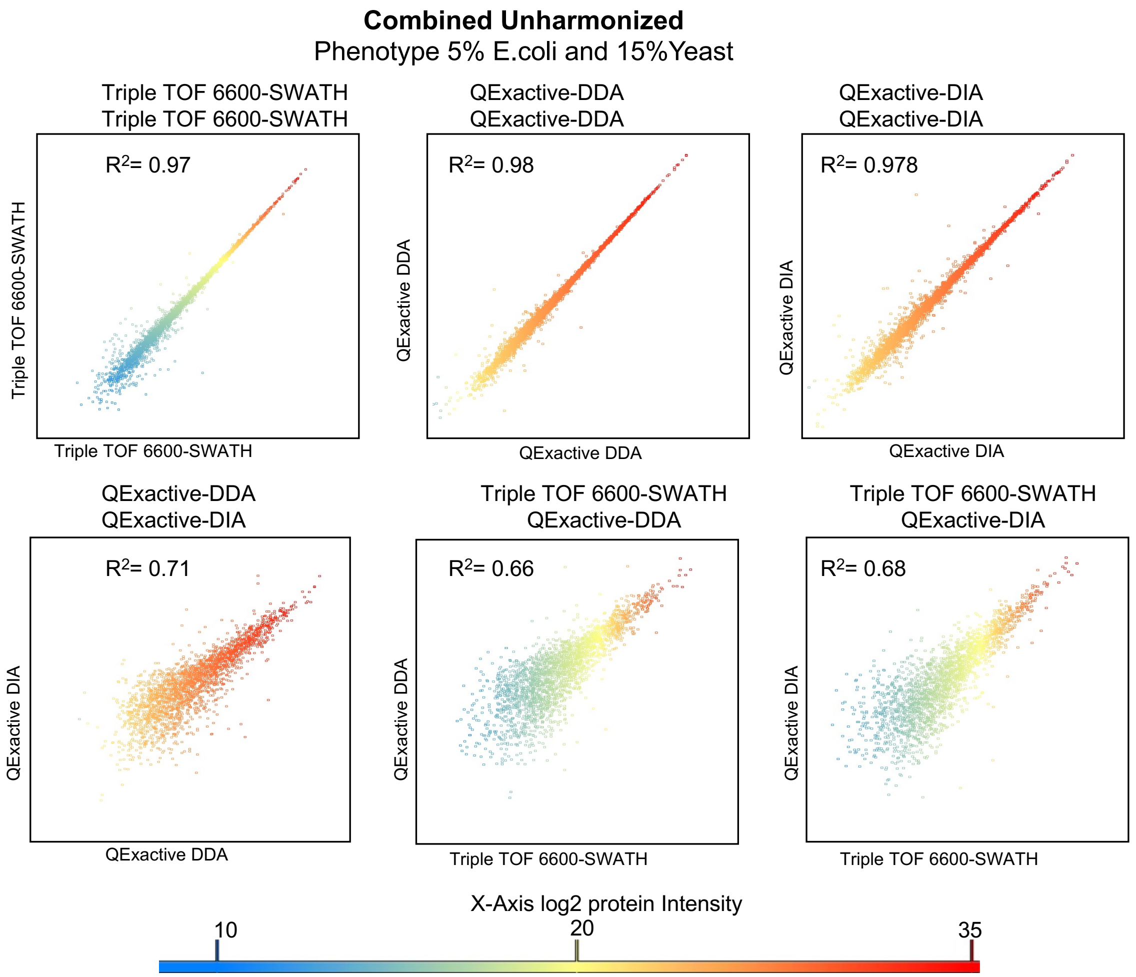

Supplementary Figure 3: Comparison of technical replicates of K562 Chronic Myelogenous Leukemia cells spiked with 5 % yeast and 15 % E. coli, within and across LC-MS/MS setups (Triple TOF6600 SWATH; QExactive DIA; QEcaxtive DDA) for unharmonized data. *Scatter plot visualization and corresponding Pearson correlation coefficient for phenotype 1 samples, measured with similar (upper panels) and different (lower panels) LC-MS/MS setups for all executed strategies for unharmonized data. Source data are provided as a Source Data file.*

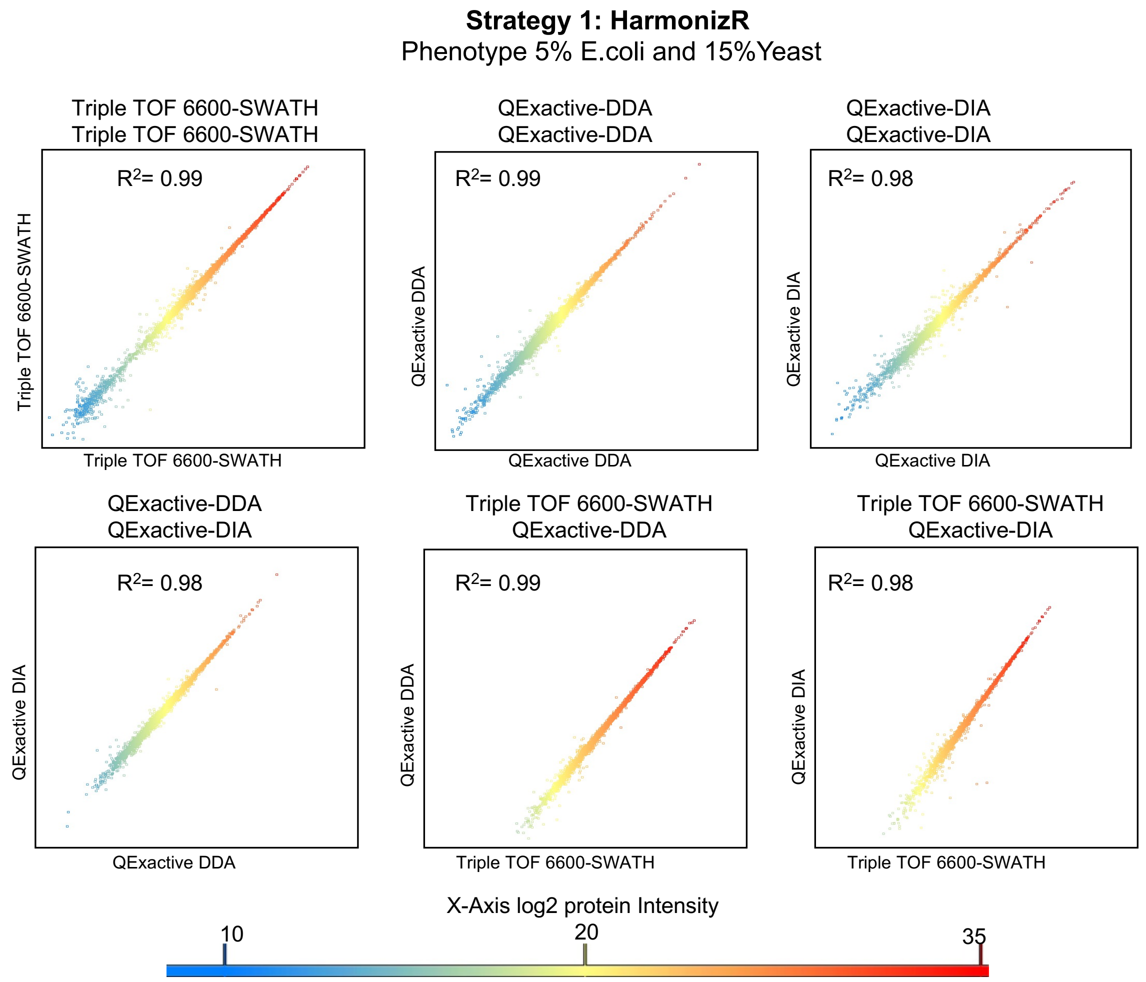

Supplementary Figure 4: Comparison of technical replicates of K562 Chronic Myelogenous Leukemia cells spiked with 5 % yeast and 15 % E. coli, within and across LC-MS/MS setups (Triple TOF6600 SWATH; QExactive DIA; QEcaxtive DDA) for HarmonizR processed data. *Scatter plot visualization and corresponding Pearson correlation coefficient for phenotype 1 samples, measured with similar (upper panels) and different (lower panels) LC-MS/MS setups for all executed strategies after HarmonizR usage. A non-parametric Bayesian framework with L/S scaling, integrated in the ComBat algorithm, was applied. Source data are provided as a Source Data file.*

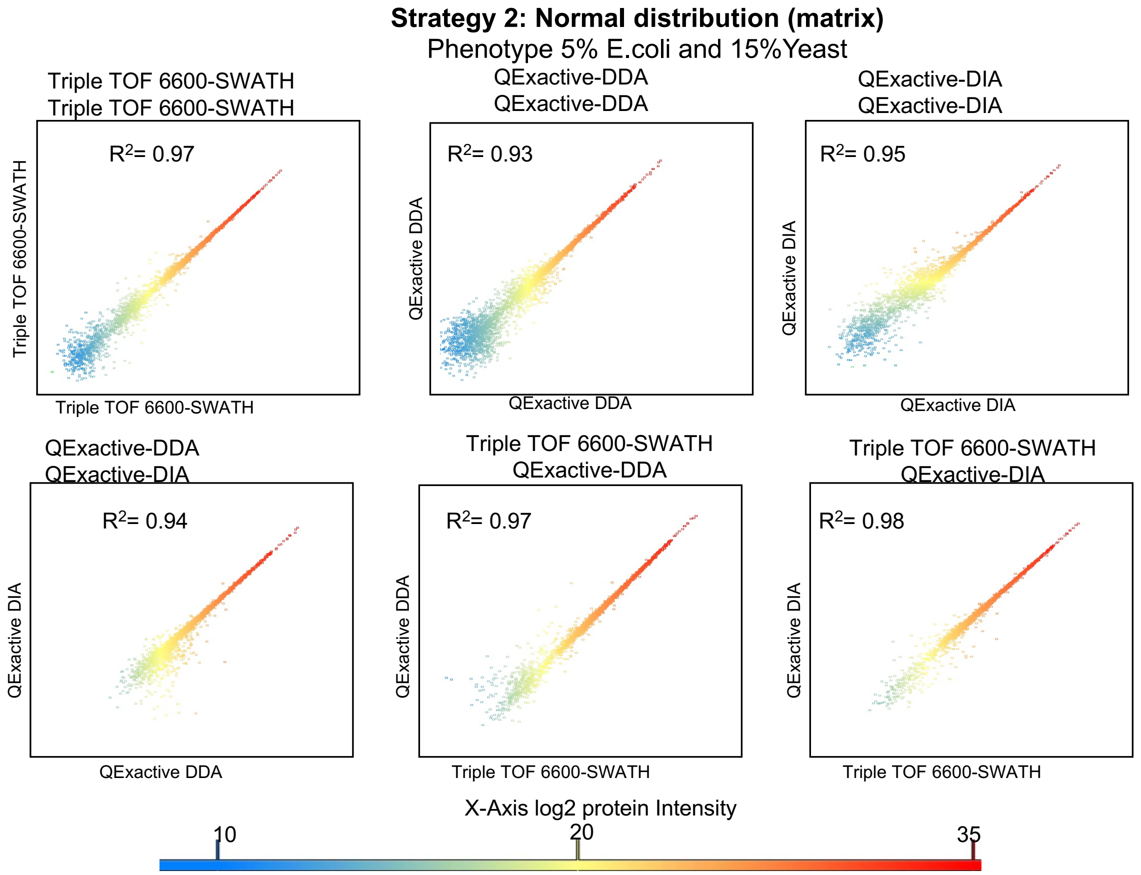

Supplementary Figure 5: Comparison of technical replicates of K562 Chronic Myelogenous Leukemia cells spiked with 5 % yeast and 15 % E. coli, within and across LC-MS/MS setups (Triple TOF6600 SWATH; QExactive DIA; QEcaxtive DDA) for column wise normal distribution imputed and ComBat processed data. *Scatter plot visualization and corresponding Pearson correlation coefficient for phenotype 1 samples, measured with similar (upper panels) and different (lower panels) LC-MS/MS setups for all executed strategies after matrix wise imputation based on the normal distribution prior to standard ComBat usage. A non-parametric Bayesian framework with L/S scaling, integrated in the ComBat algorithm, was applied. Source data are provided as a Source Data file.*

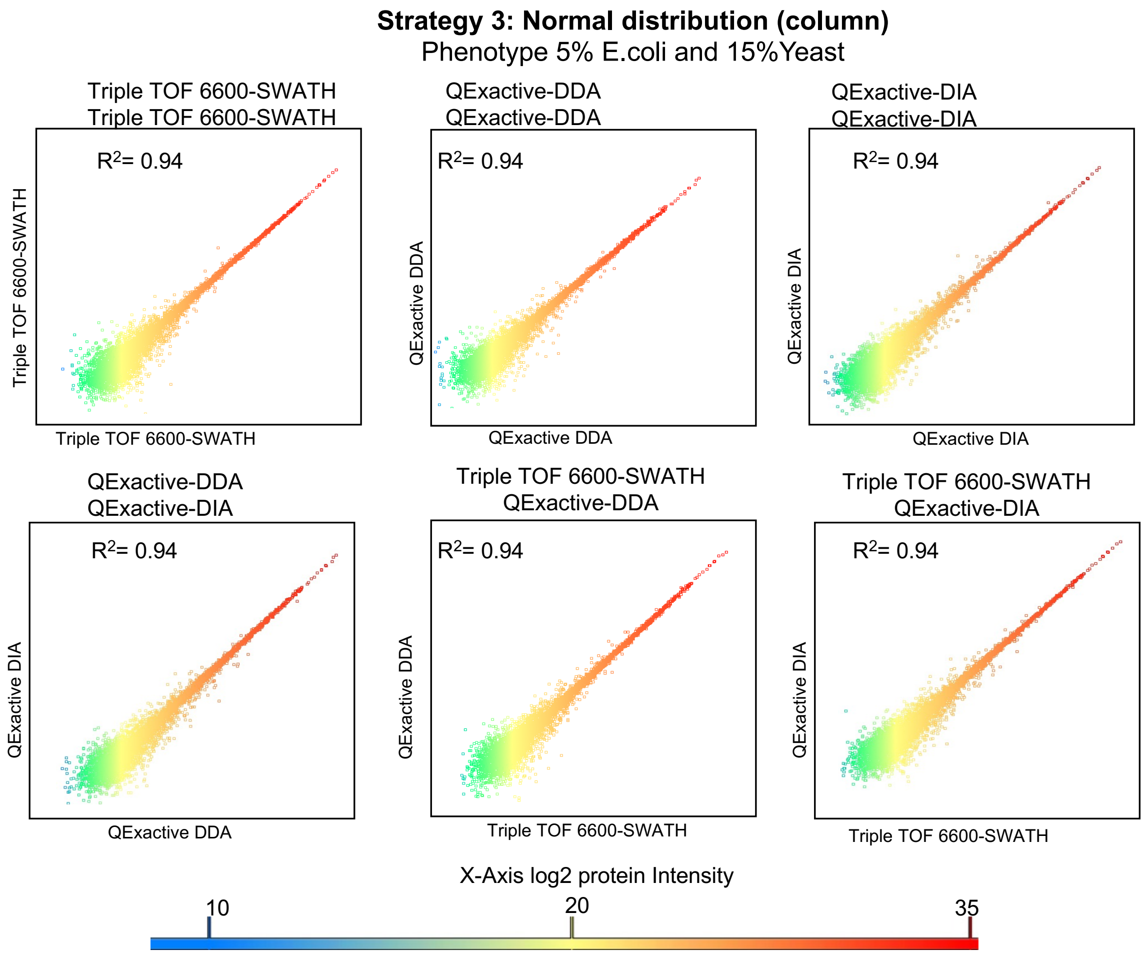

Supplementary Figure 6: Comparison of technical replicates of K562 Chronic Myelogenous Leukemia cells spiked with 5 % yeast and 15 % E. coli, within and across LC-MS/MS setups (Triple TOF6600 SWATH; QExactive DIA; QEcaxtive DDA) for matrix wise normal distribution imputed and ComBat processed data. *Scatter plot visualization and corresponding Pearson correlation coefficient for phenotype 1 samples, measured with similar (upper panels) and different (lower panels) LC-MS/MS setups for all executed strategies after column wise imputation based on the normal distribution prior to standard ComBat usage. A non-parametric Bayesian framework with L/S scaling, integrated in the ComBat algorithm, was applied. Source data are provided as a Source Data file.*

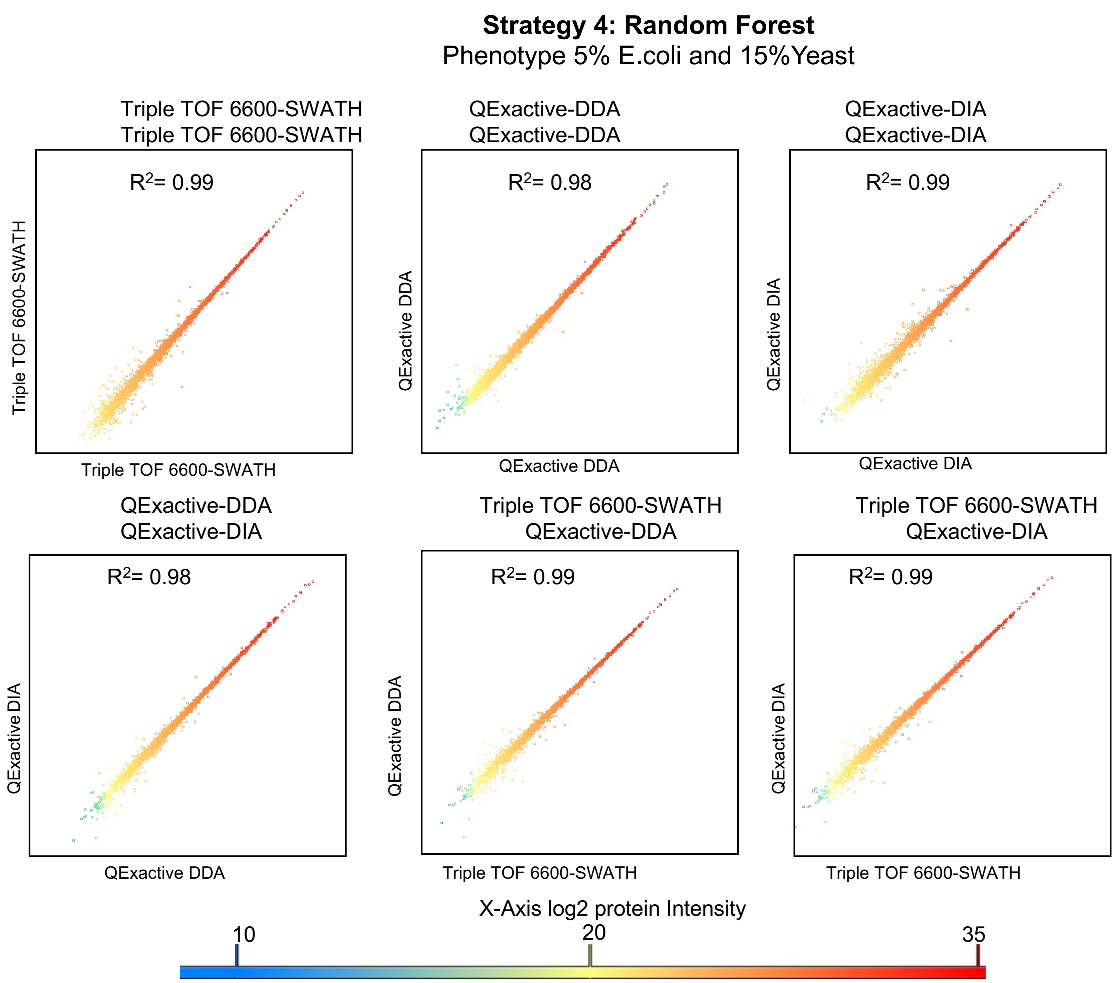

Supplementary Figure 7: Comparison of technical replicates of K562 Chronic Myelogenous Leukemia cells spiked with 5 % yeast and 15 % E. coli, within and across LC-MS/MS setups (Triple TOF6600 SWATH; QExactive DIA; QEcaxtive DDA) for random forest imputed and ComBat processed data. *Scatter plot visualization and corresponding Pearson correlation coefficient for phenotype 1 samples, measured with similar (upper panels) and different (lower panels) LC-MS/MS setups for all executed strategies after random forest imputation prior to standard ComBat usage. A non-parametric Bayesian framework with L/S scaling, integrated in the ComBat algorithm, was applied. Source data are provided as a Source Data file.*

Strategy 5: HarmonizR + Random Forest Imputation
Phenotype 5% E.coli and 15%Yeast

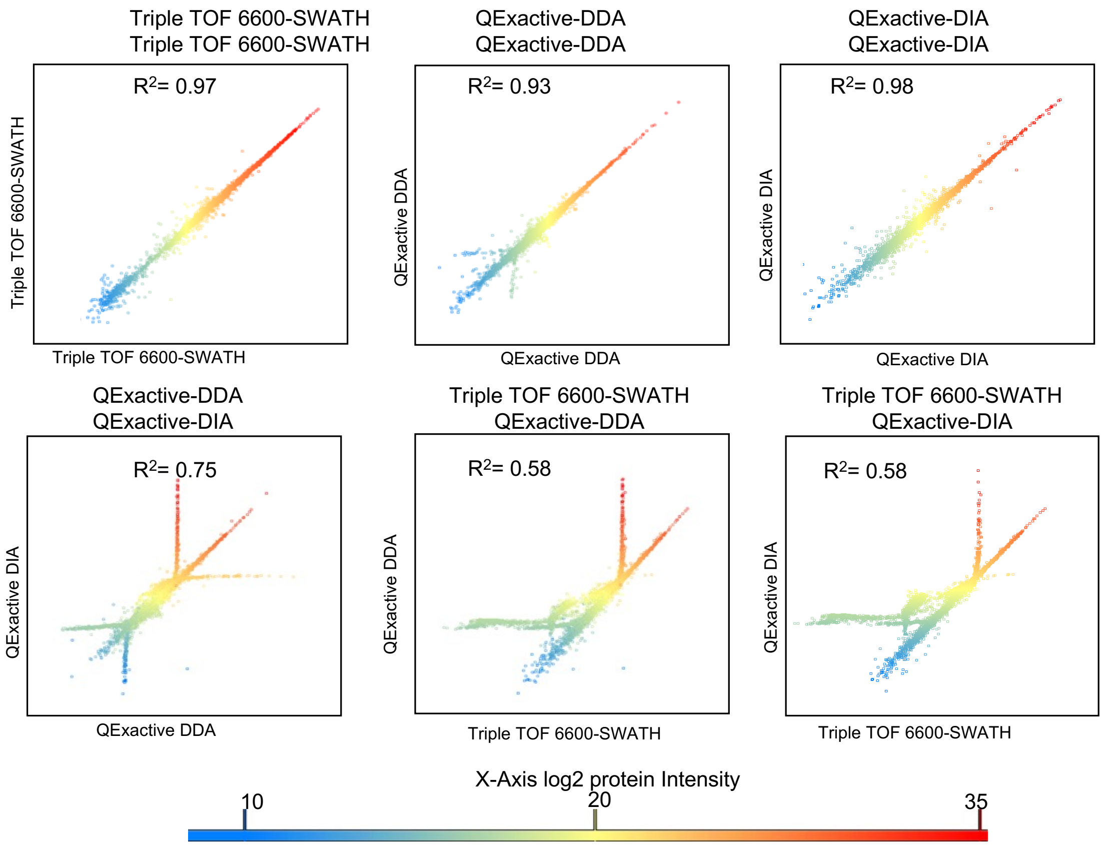

Supplementary Figure 8: Comparison of technical replicates of K562 Chronic Myelogenous Leukemia cells spiked with 5 % yeast and 15 % E. coli, within and across LC-MS/MS setups (Triple TOF6600 SWATH; QExactive DIA; QEcaxtive DDA) for HarmonizR processed and random forest imputed data. *Scatter plot visualization and corresponding correlation coefficient for phenotype 1 samples, scatter plot visualization and corresponding Pearson correlation coefficient for phenotype 1 samples, measured with similar (upper panels) and different (lower panels) LC-MS/MS setups for all executed strategies after random forest imputation and after HarmonizR usage. A non-parametric Bayesian framework with L/S scaling, integrated in the ComBat algorithm, was applied. Source data are provided as a Source Data file.*

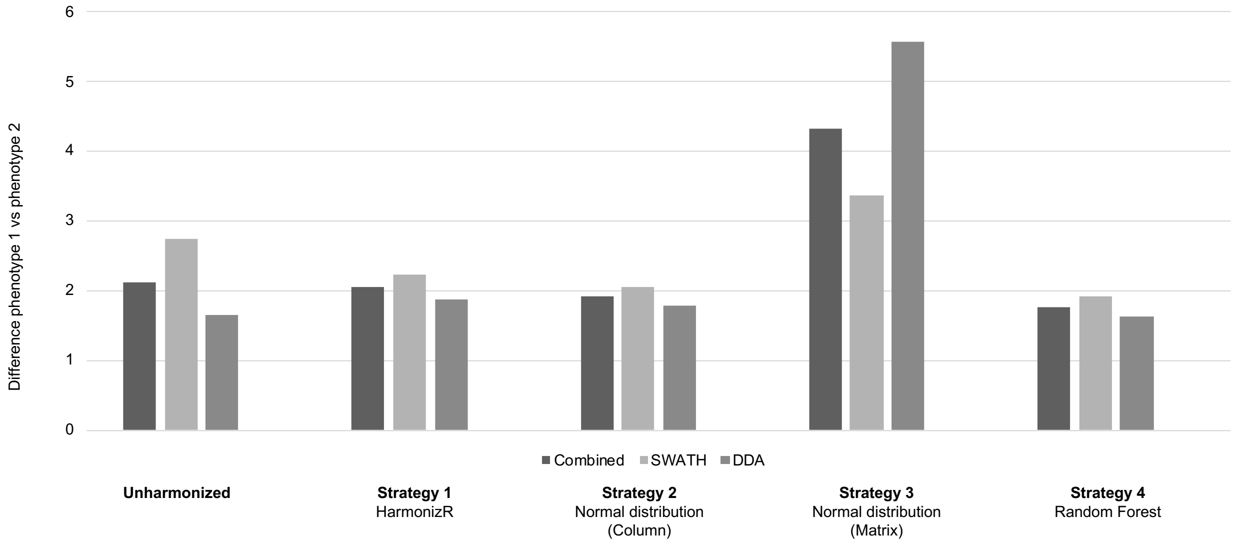

Supplementary Figure 9: Mean difference between phenotype 1: 10 % yeast 10 % E.Coli and phenotype 2: 5 % yeast and 15 % E. Coli for the Saccharomyces Cerevisiae Protein RPL16A prior to and after data harmonization with different missing value handling strategies.

Protein abundance differences after HarmonizR (ComBat, nonparametric Bayes Framework, L/S Scaling) was compared to ComBat after Random Forest imputation, ComBat after matrix wise imputation from the normal distribution and ComBat after column wise imputation from the normal distribution individually for DDA (QExactive) and SWATH (Triple TOF 6600) separately. The protein was not identified in DIA measurements. Source data are provided as a Source Data file.

		Homo Sapiens		*E.Coli*		Yeast	
		%	N	%	N	%	N
**SWATH**	True	99.8 %	1955	35.9 %	176	33.7 %	223
	False	0.2 %	4	64.1 %	314	66.3 %	439
**DDA**	True	99.9 %	3372	17.5 %	51	60.8 %	274
	False	0.1 %	3	82.5 %	241	39.2 %	177
**DIA**	True	99.9 %	2135	8.4 %	30	28.1 %	136
	False	0.1 %	3	91.6 %	328	71.9 %	348
**Combined unharmonized**	True	99.9 %	3785	9.6 %	58	36.9 %	335
	False	0.1 %	4	90.4 %	547	63.1 %	573
**Combined harmonized**	True	99.9 %	3710	9.8 %	59	45.3 %	409
	False	0.1 %	4	90.2 %	544	54.7 %	493

Supplementary Figure 10: Correctly and falsely assigned observations for expected regulations between phenotype 1: 10 % yeast 10 % E. Coli and phenotype 2: 5 % yeast and 15 % E. Coli for each individual quantification technique (DDA, DIA, SWATH), after data integration and after subsequent HarmonizR usage*. Two sample Student`s T-testing was used to determine statistically significant differential abundant proteins between phenotype 1 and 2 (p-value <0.05). For human proteins, no difference was expected. Proteins were assigned as true negative (TN) if they were identified with a p-value > 0.05 or a p-value < 0.05 and a foldchange < 1.5 between phenotype 1 and 2. Human proteins that did not match these criteria were classified as false positive (FP). E. Coli proteins identified with a p-value < 0.05 and a foldchange > 1.5 or yeast proteins identified p-value < 0.05 and a foldchange > 2 were identified as true positive (TP). E. Coli and yeast proteins that did not match these criteria were classified as false negative (FN). Source data are provided as a Source Data file.*

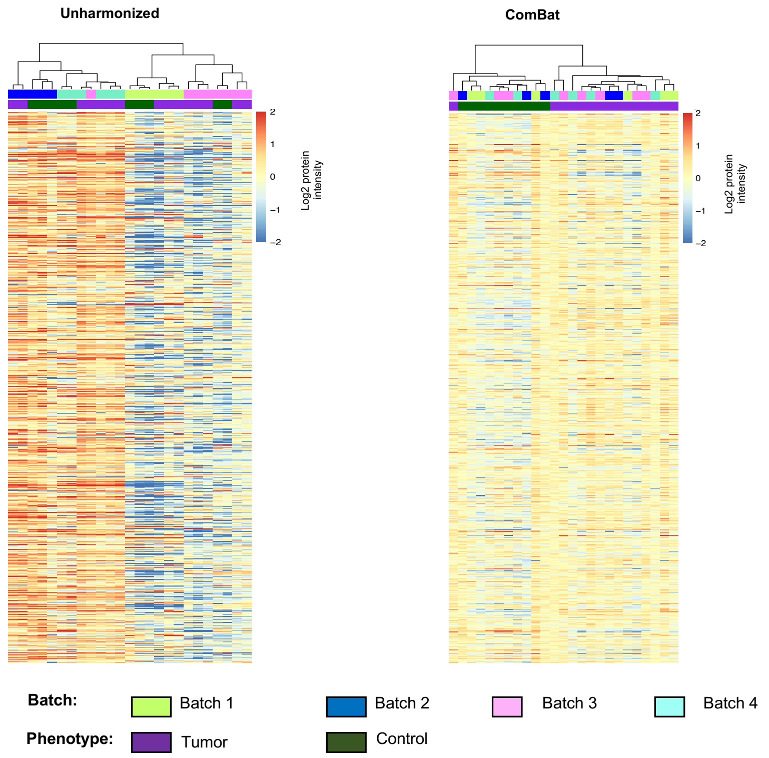

Supplementary Figure 11: Comparison of Limma and ComBat for data harmonization **across different time points and tissue preservations, based on cerebellar tumors of hGFAP-cre: SmoM2$^{Fl/+}$ and cerebella controls of littermates.** *Heatmap visualization of Pearson correlation-based hierarchical clustering with Ward.D linkage for each tissue type and timepoint for unharmonized combined data, after limma (removeBatchEffect()) and L/S scaling based parametric ComBat, based on 1002 proteins found in all batches. Source data are provided as a Source Data file.*

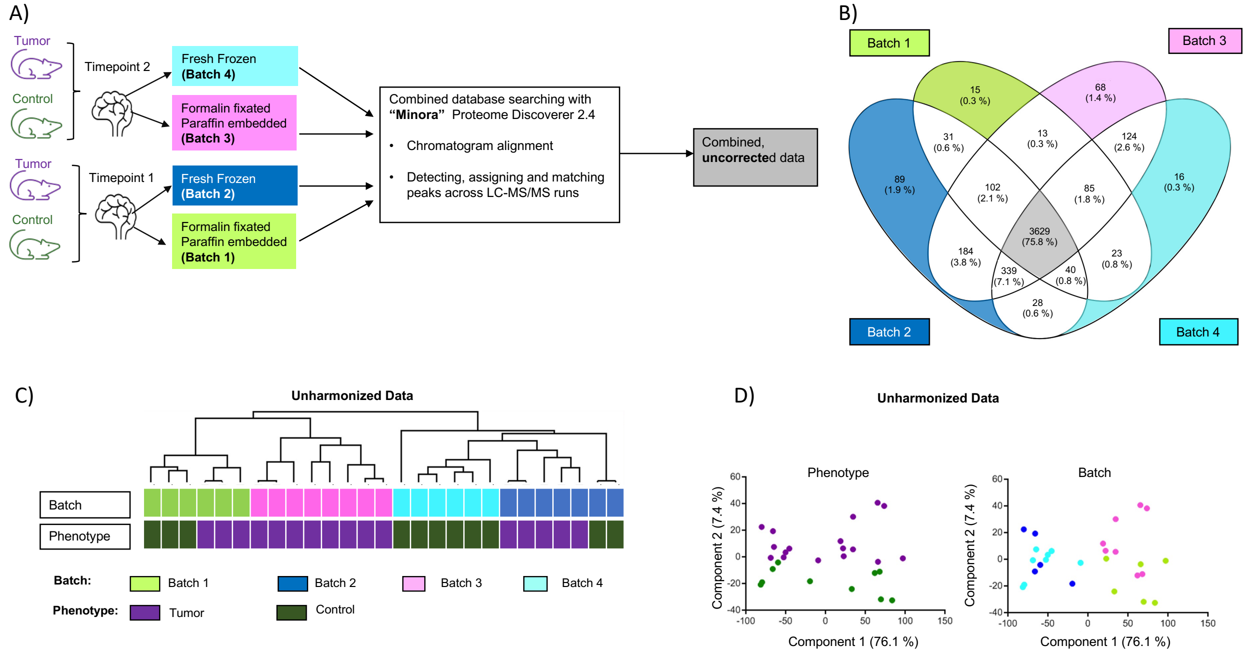

Supplementary Figure 12: HarmonizR application to the batch effect reduction across different time points and tissue preservations, based on cerebellar tumors of hGFAP-cre:SmoM2$^{Fl/+}$ and cerebella controls of littermates after optimized database searching. *A) Schematic overview. LC-MS/MS data from FFPE and FF tissue of cerebellar hGFAP-cre::SmoM2$^{Fl/+}$ tumors and cerebellar controls of littermates at two different analysis timepoints. Spectra were submitted to combined database search, using the Sequest algorithm, implemented in Proteome Discoverer 2.4. To rescue low abundant signals and reduce non-biological variance across LC-MS/MS measurements, the Minora feature detector was enabled. B) Batch count distribution of all 4786 proteins quantified at least 2 times in a batch. C) Pearson correlation-based hierarchical clustering with Ward.D linkage for each tissue type and timepoint for unharmonized combined data after combined database search. D) Scatter plot visualization of the first two principal components in non-linear iterative squares (NIPALS) PCA based on 3564 proteins quantified in at least 50 % of all samples.*

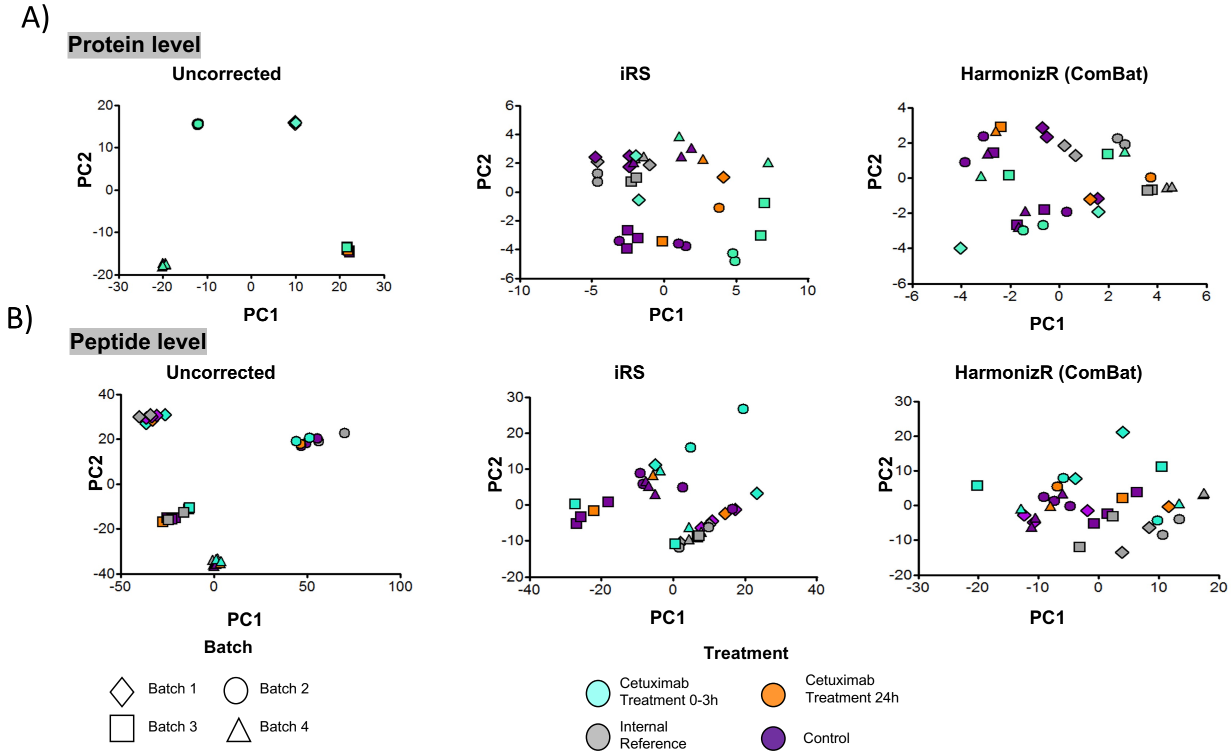

Supplementary Figure 13: Visualization of the first two principal components (PC), comparing HarmonizR and iRS for batch effect reduction in Multiplex TMT experiments for Cetuximab treated HiFi cells at the peptide and protein level. *A) Scatter plot distribution of samples across PC1 and PC2 in NIPALS-PCA, based on 8877 peptides found in 50 % of all samples for unharmonized data, after iRS normalization and after HarmonizR (ComBat). B) Scatter plot distribution of samples across PC1 and PC2 in NIPALS-PCA, based on 2152 proteins found in 50 % of all samples for unharmonized data, after iRS normalization and after HarmonizR (ComBat). Data reprocessed from Stepath et al. (2020). Source data are provided as a Source Data file.*

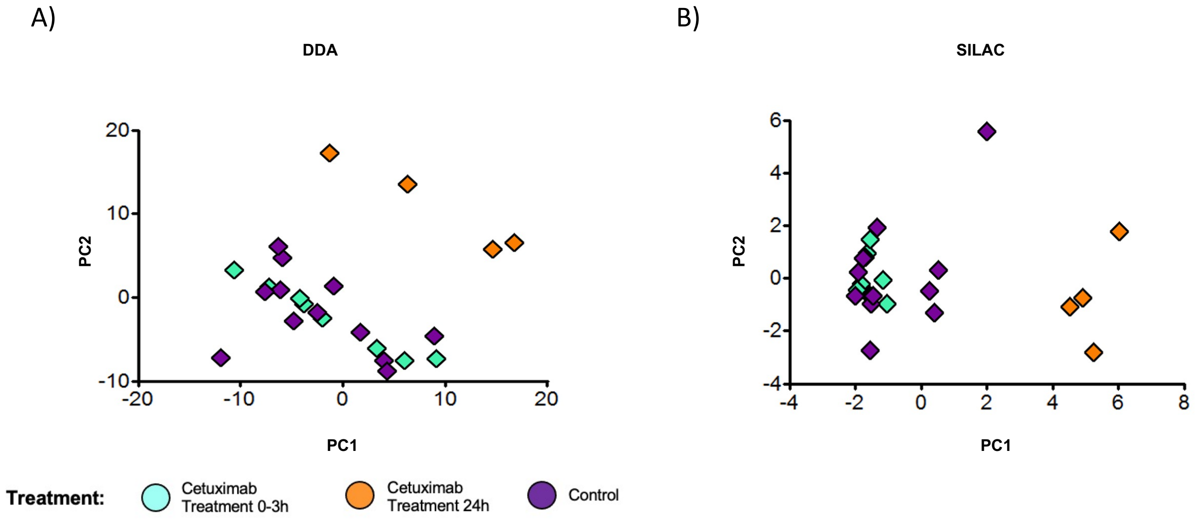

Supplementary Figure 14: Visualization of the First two principal components (PC) for DDA and SILAC experiments on Cetuximab treated HiFi cells at the protein level. *Scatter plot distribution of samples across PC1 and PC2 in NIPALS-PCA, A) based on 3903 proteins, found 50 % of all HiFi cells, measured using data dependent acquisition (DDA) mode Label Free quantification (LFQ), B) based on 1750 proteins, found 50 % of all HiFi cells, measured using stable isotope labeling by amino acids in cell culture (SILAC). Data reprocessed from Stepath et al. (2020) . Source data are provided as a Source Data file.*

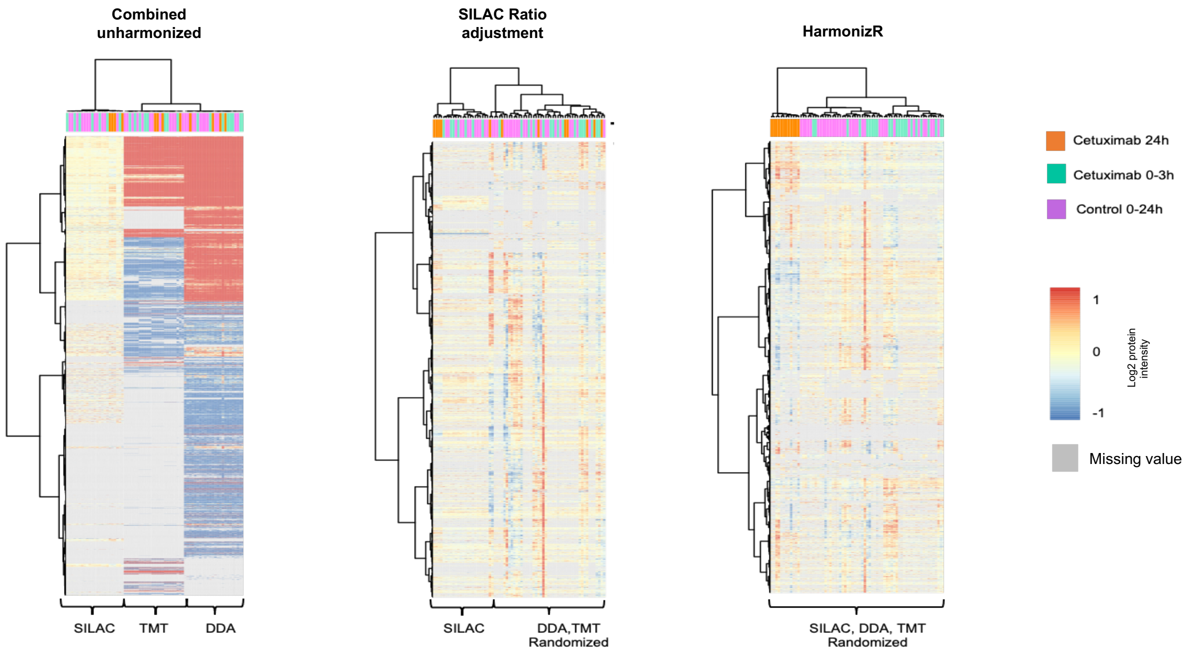

Supplementary Figure 15: Comparison of unharmonized data, SILAC ratio adjusted data and HarmonizR processed data for data integration between SILAC, TMT and DDA data. *Heatmap visualization of Pearson correlation-based hierarchical clustering with Ward.D linkage for all proteins considerable for unharmonized data, after SILAC ratio adjustment and data after HarmonizR adjustment. A non-parametric Bayesian framework with L/S scaling, integrated in the ComBat algorithm, was applied. Source data are provided as a Source Data file.*
