# Supplementary material for: HarmonizR enables data harmonization across independent proteomic datasets with appropriate handling of missing values
Source: Nat Commun. 2022 Jun 20;13:3523. doi: 10.1038/s41467-022-31007-x (PMC9209422; doi:10.1038/s41467-022-31007-x)
Supplement: Supplementary file 2 — Reporting Summary [file 41467_2022_31007_MOESM2_ESM.pdf]

nature portfolio

Corresponding author(s): Dr. Christoph Krisp
Prof. Julia E. Neumann

Last updated by author(s): May 12, 2022

Reporting Summary

Nature Portfolio wishes to improve the reproducibility of the work that we publish. This form provides structure for consistency and transparency in reporting. For further information on Nature Portfolio policies, see our Editorial Policies and the Editorial Policy Checklist.

Statistics

For all statistical analyses, confirm that the following items are present in the figure legend, table legend, main text, or Methods section.

n/a	Confirmed	
☐	☒	The exact sample size (n) for each experimental group/condition, given as a discrete number and unit of measurement
☐	☒	A statement on whether measurements were taken from distinct samples or whether the same sample was measured repeatedly
☐	☒	The statistical test(s) used AND whether they are one- or two-sided<br>*Only common tests should be described solely by name; describe more complex techniques in the Methods section.*
☐	☒	A description of all covariates tested
☐	☒	A description of any assumptions or corrections, such as tests of normality and adjustment for multiple comparisons
☐	☒	A full description of the statistical parameters including central tendency (e.g. means) or other basic estimates (e.g. regression coefficient) AND variation (e.g. standard deviation) or associated estimates of uncertainty (e.g. confidence intervals)
☐	☒	For null hypothesis testing, the test statistic (e.g. F, t, r) with confidence intervals, effect sizes, degrees of freedom and P value noted<br>*Give P values as exact values whenever suitable.*
☒	☐	For Bayesian analysis, information on the choice of priors and Markov chain Monte Carlo settings
☐	☒	For hierarchical and complex designs, identification of the appropriate level for tests and full reporting of outcomes
☐	☒	Estimates of effect sizes (e.g. Cohen's d, Pearson's r), indicating how they were calculated

Our web collection on statistics for biologists contains articles on many of the points above.

Software and code

Policy information about availability of computer code

Data collection	No Software was used

Data analysis	Data harmonization was performed using the custom code HarmonizR (accessible via: https://github.com/SimonSchlumbohm/HarmonizR and Zendo DOI: 10.5281/zenodo.6553171). This algorithm is a framework around the ComBat and Limma algorithms for Batch effect correction (published in 2007 by Johnson et al. and published in 2015 by Ritchie et al. respectively), that are part of the SVA software package in the R software environment DOI: 10.18129/B9.bioc.sva). Further used R packages are: mixOmics (DOI: 10.18129/B9.bioc.mixOmics; Version 6.20.0), and pheatmap ((Version 1.0.12) , accessible via the CRAN project (https://cran.r-project.org/web/packages/pheatmap/index.html). Database searching from raw LC-MS/MS spectra was performed using MaxQuant software (Max Plank Institute for Biochemistry, Version 1.6.2.10) or Proteome Discoverer(Thermo Fisher Scientific, Version 2.4.), respectively. Study specific normalization and t-testing was performed in Perseus (Max Plank Institute for Biochemistry, Version 1.6.15.0). Pearson correlation coefficients, were calculated and visualized using GraphPad Prism (Version 5). Abundance distributions of individual proteins were visualized using Microsoft Excel in the version 16.5. Boxplots were generated, using the in-build boxplot () function in R 4.0.4.The visualization of the speedup has been done in the Python programming language (Version 3.8.) using the “matplotlib” package (Version 3.5.2.). Venn diagrams were generated using Venny (BioinfoGP, Version 2.1.0).<br>Required software versions for the HarmonizR program listed below:<br>R >= 4.0.4<br>SVA >= 3.36.0<br>doParallel >= 1.0.16<br>foreach >= 1.5.1<br>janitor >= 2.1.0<br>plyr >= 1.8.6

For manuscripts utilizing custom algorithms or software that are central to the research but not yet described in published literature, software must be made available to editors and reviewers. We strongly encourage code deposition in a community repository (e.g. GitHub). See the Nature Portfolio guidelines for submitting code & software for further information.

Data

Policy information about availability of data

All manuscripts must include a data availability statement. This statement should provide the following information, where applicable:

- Accession codes, unique identifiers, or web links for publicly available datasets
- A description of any restrictions on data availability
- For clinical datasets or third party data, please ensure that the statement adheres to our policy

All raw and processed proteomic data generated in this study has been deposited to ProteomeXchange Consortium via PRIDE, under the accession code PXD027467 (https://www.ebi.ac.uk/pride/archive/projects/PXD027467). Publicly available datasets, used in this study, can be accessed via PRIDE, under the accession code PXD014565 (Stepath et al.20 (https://pubs.acs.org/doi/full/10.1021/acs.jproteome.9b00701)) (https://www.ebi.ac.uk/pride/archive/projects/PXD014565) or through the Clinical Proteomic Tumor Analysis Consortium Data Portal (https://cptac-data-portal.georgetown.edu/cptacPublic/) under the accession code PDC000204 (Petralia et al. 23 (https://pubmed.ncbi.nlm.nih.gov/33242424/)), respectively.

Field-specific reporting

Please select the one below that is the best fit for your research. If you are not sure, read the appropriate sections before making your selection.

☒ Life sciences ☐ Behavioural & social sciences ☐ Ecological, evolutionary & environmental sciences

For a reference copy of the document with all sections, see nature.com/documents/nr-reporting-summary-flat.pdf

Life sciences study design

All studies must disclose on these points even when the disclosure is negative.

Sample size	To initially test for the applicability of data harmonizations between different mass spectrometers and label free quantification methods six samples were measured with different experimental setups. Three technical replicates of 80% human cell digests, 10% E. Coli and 10% yeast and three technical replicates with 80% human, 15% E. Coli and 5% yeast were analyzed. To evaluate the ability of ComBat HarmonizR to harmonize between different tissue types and preparation time-points a total number of 25 samples was analyzed. 15 were classified as tumor samples . 10 were cerebellar controls. For the data harmonization between different quantification approaches 25 samples were analyzed with each method of DDA based label free quantification, SILAC and TMT. For TMT measurements additionally 8 internal reference samples were measured. For the TMT dataset published by Petralia et al. 230 samples from eight different pediatric brain tumor entities were analyzed.
Data exclusions	No data was excluded
Replication	All in house experiments were performed at least in biological triplicates. For publicly available datasets the availability and number of replicates depended on the respective experimental setup given in the original study.
Randomization	Phenotypes were randomized for each individual experimental setup
Blinding	Blinding was not required, as the data harmonization between defined experimental setups was the aim of this study

Reporting for specific materials, systems and methods

We require information from authors about some types of materials, experimental systems and methods used in many studies. Here, indicate whether each material, system or method listed is relevant to your study. If you are not sure if a list item applies to your research, read the appropriate section before selecting a response.

Materials & experimental systems

n/a	Involved in the study
x	Antibodies
	x Eukaryotic cell lines
x	Palaeontology and archaeology
	x Animals and other organisms
x	Human research participants
x	Clinical data
x	Dual use research of concern

Methods

n/a	Involved in the study
x	ChIP-seq
x	Flow cytometry
x	MRI-based neuroimaging

Eukaryotic cell lines

Policy information about cell lines

Cell line source(s)	K562 Chronic Myelogenous Leukemia ccells were purchased from Promega (Madison).
Authentication	No cell line was Authenticated
Mycoplasma contamination	No Mycoplasma contamination was detected
Commonly misidentified lines (See ICLAC register)	No commonly misidentified cell line was used

Animals and other organisms

Policy information about studies involving animals; ARRIVE guidelines recommended for reporting animal research

Laboratory animals	hGFAP-cre mice were published before (PMID: 11668683). SmoM2Fl/Fl mice were published before (PMID: 17047082) Both lines were purchased from The Jackson Laboratories (Bar Harbor, ME, USA) and bred with a C57BL/6J background in specific pathogen free environment with temperatures between 22 ± 1 °C, humidity between 45 -65 % and a dark/light cycle changing every 12 hours. Both male and female mice were used. Tumors and cerebella were analyzed at postnatal day 13.
Wild animals	No wild animals were used
Field-collected samples	No field-collected samples were used
Ethics oversight	All experiments using animals were approved by the local animal care committee (Behörde für Justiz und Verbraucherschutz in Hamburg, TVA N99/2019) and handling was conducted in accordance with local governmental and institutional animal care regulations.

Note that full information on the approval of the study protocol must also be provided in the manuscript.
